# Beneficial fungal root endophyte *Piriformospora indica* inhibits bitter gourd mosaic complex disease incited by combined infection of tomato leaf curl, papaya ringspot, and cucumber mosaic viruses without compromising crop growth and yield by orchestrating ROS production and scavenging through retrograde signaling

**DOI:** 10.3389/fmicb.2026.1781341

**Published:** 2026-03-18

**Authors:** Deepa R. Chandran, Joy Michal Johnson, A. Mary Sharmila, Saru Sara Sam, T. Anuradha, S. Sarada, K. B. Soni, K. D. Prathapan

**Affiliations:** 1 Department of Plant Pathology, College of Agriculture, Vellayani, Kerala Agricultural University, Thiruvananthapuram, Kerala, India; 2 Department of Molecular Biology and Biotechnology, College of Agriculture, Vellayani, Kerala Agricultural University, Thiruvananthapuram, Kerala, India; 3 Department of Vegetable Science, College of Agriculture, Vellayani, Kerala Agricultural University, Thiruvananthapuram, Kerala, India; 4 Department of Entomology, College of Agriculture, Vellayani, Kerala Agricultural University, Thiruvananthapuram, Kerala, India

**Keywords:** antioxidants, bitter gourd mosaic complex, cucumber mosaic virus, papaya ringspot virus, *Piriformospora indica*, reactive oxygen species, retrograde signaling, tomato leaf curl virus

## Abstract

Bitter gourd mosaic complex (BGMC)—caused by the combined infection of tomato leaf curl virus (ToLCV), papaya ringspot virus (PRSV), and cucumber mosaic virus (CMV)—poses a major threat to bitter gourd (*Momordica charantia* var. *charantia* L.) cultivation, resulting in severe yield losses. In this study, *Piriformospora indica*, a beneficial fungal root endophyte, was evaluated for its potential in managing BGMC. BGMC produced a wide range of symptoms. The presence of ToLCV, PRSV, and CMV in BGMC was confirmed serologically and molecularly. *Piriformospora indica* colonization in bitter gourd plants significantly reduced BGMC incidence from 100% to less than 20%, and the disease severity from 84.17 to 9.02%, when the viruses were inoculated by wedge grafting at 15 days after the colonization. Further, *P. indica* drastically reduced the disease severity from >75% to <30% under field conditions. Surprisingly, the fungus could significantly inhibit all three viruses responsible for BGMC, as evidenced by the repression of their coat protein genes and the virus titers in DAS-ELISA and PCR/RT-PCR. *P. indica*-colonization also enhanced growth and fruit yield. Biochemical analyses demonstrated that *P. indica*-colonization significantly suppressed ROS and H_2_O_2_ accumulation in the viruses-infected plants, while markedly enhancing the activities of antioxidant enzymes such as catalase, superoxide dismutase, and peroxidase (> 5-fold). Moreover, the concomitant downregulation of ROS and H_2_O_2_ marker genes (up to 12-fold less) and upregulation of antioxidant genes (up to 10-fold) located in the nucleus (*WRKY40*, *MYB51*, *CML37*, *AGP5*, *CSD1*, *APX1*), chloroplast (*LOX2*, *PTOX*, *FSD1*, *FSD2*), and mitochondria (*HSPRO1*, *DIC2*, *PRX*, *MSD1*, *AOX2*) of leaves clearly indicate the role of *P. indica* in orchestrating the regulation of ROS production and its scavenging through systemic retrograde signaling molecule(s) produced by the root endophyte. This premier study highlights the inherent ability of *P. indica* to diminish the complex symptoms of BGMC by simultaneous inhibition of ToLCV, PRSV, and CMV. ROS and H_2_O_2_-producing and scavenging genes located in the nucleus, chloroplast, and mitochondria are modulated by the retrograde signaling molecule(s) produced during the multipartite interaction involving the endophyte, plant, and the viruses.

## 1 Introduction

Bitter gourd (*Momordica charantia* var. *charantia* L.), also known as bitter melon, is a high-value cucurbit cultivated throughout tropical and subtropical regions for its nutritional and pharmacological benefits, including antidiabetic, antioxidant, and antimicrobial properties (Fuangchan et al., 2011; Sur and Ray, 2020; Behera et al., 2023). Despite its economic and therapeutic importance, bitter gourd production is significantly hampered by multiple virus infections, particularly mixed infections of tomato leaf curl virus (ToLCV), papaya ringspot virus (PRSV), and cucumber mosaic virus (CMV) (Nagarajan and Ramakrishnan, 1971; Rajinimala et al., 2005; Nagendran et al., 2017; Radhika and Umamaheswaran, 2017; Kumari et al., 2021; Krishnan et al., 2022b; Li et al., 2022; Mulholland et al., 2023; Chandran et al., 2024), in addition to bitter gourd distortion mosaic virus (BDMV) (Tiwari et al., 2010; Naik et al., 2022). The above-mentioned viruses cause bitter gourd mosaic complex (BGMC) disease, which poses a major threat to bitter gourd cultivation worldwide. These viruses, transmitted primarily through seeds and secondarily by whiteflies and aphids, induce severe leaf curling, mottling, vein clearing, stunting, and fruit malformation, leading to substantial yield losses (Nagendran et al., 2014; Li et al., 2022; Gomathidevi et al., 2023; Chandran et al., 2024). In recent years, BGMC disease has resulted in 100% crop loss in India, China, and Australia (Kumari et al., 2021; Li et al., 2022; Mulholland et al., 2023; Chandran et al., 2024). Conventional management approaches such as vector control using insecticides, crop rotation, and the deployment of resistant cultivars have shown limited effectiveness due to the rapid evolution of viral strains and vector populations, underscoring the need for sustainable disease management strategies.

Beneficial fungal root endophytes have emerged as promising biocontrol and plant growth-promoting agents capable of enhancing host tolerance against biotic and abiotic stresses (Oelmüller et al., 2009; Johnson, 2014; Gill et al., 2016). *Piriformospora indica*, a root-colonizing basidiomycete of the Sebacinales, establishes a mutualistic symbiosis with a wide range of plants, improving nutrient acquisition, growth, and yield (Varma et al., 2012). In addition to growth promotion, *P. indica* modulates host defense responses through systemic signaling, including regulation of cytosolic calcium, reactive oxygen species (ROS), phytohormones, and antioxidant enzymes (Johnson et al., 2014a,b; Matsuo et al., 2015; Xu et al., 2017). The ability of *P. indica* to fine-tune oxidative stress pathways makes it a strong candidate for protecting plants against oxidative damage triggered by pathogens.

Plants constantly produce ROS and H_2_O_2_ in chloroplasts, mitochondria, peroxisomes, cytoplasm, nucleus, and other sites of the cell due to photosynthesis, respiration, and other metabolic processes (Mittler et al., 2004; Tripathy and Oelmüller, 2012). Plant pathogens, including fungi, bacteria, phytoplasma, and viruses, produce different types of symptoms by inducing excess ROS and H_2_O_2_, leading to their accumulation in plants (Torres, 2010; Matsuo et al., 2015). Plant viruses cause discoloration of leaves, such as mosaic, mottling, vein clearing, vein banding, and yellow spots, which subsequently result in rosetting and stunting of plants (Hull, 2014; Chandran et al., 2024). Such discoloration symptoms are produced due to the excess production and accumulation of ROS and H_2_O_2_, which further enhance chlorophyll degradation besides inhibiting chlorophyll biosynthesis (Apel and Hirt, 2004; Miller et al., 2010; Sinijadas, 2024). The antioxidant enzymes and antioxidants detoxify the ROS and stabilize the redox state in different cellular organelles (Mittler et al., 2004; Dietz et al., 2006; Mehterov et al., 2012). Viral infections are known to induce chloroplast dysfunction and ROS overproduction, which play a dual role as both defense signals and mediators of cell death (Chandran, 2025). Excess ROS accumulation, combined with reduced antioxidant capacity, is a hallmark of virus-induced symptom expression and cytotoxicity (Krishnan et al., 2022a; Chandran, 2025). Managing viral diseases, therefore, often depends on restoring redox balance, enhancing antioxidant defenses, and maintaining photosynthetic efficiency. Previous studies have demonstrated that *P. indica* colonization can reprogram host transcriptional and metabolic networks, including redox regulation, to confer systemic tolerance against biotic stress (Johnson, 2014; Matsuo et al., 2015; Krishnan et al., 2022a).

In this study, we investigated whether *P. indica* colonization can mitigate bitter gourd mosaic complex disease caused by the combined infection of ToLCV, PRSV, and CMV. We assessed symptom severity, virus titer, ROS and H_2_O_2_ accumulation, antioxidant enzyme activities, and redox-related gene expressions in the nucleus, chloroplast, and mitochondria to elucidate the mechanisms underlying the endophytic fungus-mediated protection against multiple virus infections. Our findings reveal that *P. indica* not only reduces the severity of symptoms and viral titers but also enhances antioxidant enzymes to scavenge the excess ROS and H_2_O_2_ produced and accumulated due to the multiple virus infections. Further, the endophyte modulates retrograde signaling between the nucleus, chloroplast, and mitochondria to effect the systemic antiviral response in the multipartite interaction involving the endophyte, plant, and multiple viruses. This work provides novel insights into how *P. indica* fine-tunes redox homeostasis to suppress BGMC, offering a sustainable and ecologically viable management approach for bitter gourd cultivation.

## 2 Materials and methods

### 2.1 Maintenance and mass-multiplication of *P. indica*

The *P. indica* culture maintained at the Department of Plant Pathology, College of Agriculture, Vellayani (No. INBA3202001787) was used in the studies. A 5 mm disc of *P. indica* culture was sub-cultured in potato dextrose agar (PDA) medium (pH 6.5) and incubated for 3 weeks at room temperature (27 ± 2 °C). The culture discs from these plates were transferred into 100 mL of potato dextrose broth (PDB; pH 6.5) prepared in a 250 mL conical flask (three discs per flask) and kept in an orbital shaker at 40 rpm for 3 weeks at room temperature. This broth was used as the source of *P. indica* for mass multiplication.

*Piriformospora indica* was mass-multiplied in a mixture of coco-peat, finely powdered and dried farmyard manure, and gram flour (1:1:0.02), following the protocol of Jojy et al. (2020). Coco-peat blocks were soaked in water overnight and drained the following day. The soaking and draining procedure was repeated thrice, and the coco-peat was shade-dried. To 1 kg of partially dried coco-peat, an equal quantity of dried and powdered farmyard manure and 20 g of gram flour were added, thoroughly mixed, and moistened to field capacity. This mixture was packed in polypropylene bags and autoclaved at 121 °C, 15 psi for 2 h for three consecutive days.

For mass multiplication of *P. indica*, plastic trays (42 cm x 30 cm x 9 cm) surface-sterilized with 70 percent ethanol were used. The autoclaved mixture (1 kg) was set in the tray as a layer. Three-week-old *P. indica* broth culture containing 1% of the fungal mycelia (w/w) was poured into the sterilized mixture (1 kg) and thoroughly mixed. Moisture was maintained to field capacity (35% w/w) using sterile water. The inoculated mixture was spread evenly as a layer of 4–5 cm thickness in the surface-sterilized plastic tray. The trays were then covered using sterile cling film and incubated at room temperature. The same mixture without *P. indica* was used as potting mixture for growing the control plants.

### 2.2 Co-cultivation of *P. indica* with bitter gourd seedlings

Once the mycelial run was complete (10 days), the pro-trays were filled with the mixture. The control pro-trays were filled with a mixture without *P. indica*. Seeds of the bitter gourd var. Preethi were surface-sterilized in 1% sodium hypochlorite solution for 1 min, followed by washing thrice in sterile water for 5 min each, and then soaked in sterile water overnight. The seeds were sown in pro-trays filled with *P. indica*-multiplied and control potting mixtures. The trays were kept in an insect-proof chamber under temperature (27 ± 2 °C) and humidity (85 ± 2%)-controlled conditions for uniform germination and growth.

The roots of the seedlings were examined for *P. indica* colonization. The seedlings were uprooted at weekly intervals and cleaned in tap water to dislodge the mixture clumps adhering to the roots. The cleaned roots were cut into pieces of 1 cm in length and soaked in freshly prepared 10% KOH solution (w/v) in a test tube. The test tubes with roots in KOH solution were placed in a water bath at 65 °C until the root bits softened (8 to 10 min). The root bits were taken out carefully with a brush to avoid damage and washed thrice in sterile water to remove KOH residue. These root bits were transferred into fresh test tubes containing 1 N HCl and incubated for 2 min at room temperature. To stain the fungus colonization, the root bits were washed in sterile water and placed in lactophenol trypan blue dye solution for 1 min. Microscopic slides were prepared using the stained roots, and excess stain was removed using blotting paper. The slides were observed under a compound microscope (Leica-ICC50 HD, USA) to examine the endophytic colonization of *P. indica*. The *P. indica* colonization in the roots of bitter gourd plants was further confirmed molecularly using the Pi-tef primer (*Pi-tef*-F: TCGTCGCTGTCAACAAGATG; *Pi-tef*-R: GAGGGCTCGAGCATGTTGT) (Bütehorn et al., 2000).

### 2.3 Collection of the diseased samples, symptomatology and detection of the viruses

Samples showing symptoms of BGMC were collected from different fields and observed for the nature and type of symptoms. General as well as specific symptoms of the viral disease were recorded. The diseased samples were freeze-dried in liquid nitrogen and used for the detection of the viruses, and a part was stored at −80 °C for further studies.

#### 2.3.1 Immunological detection of the viruses

The diseased samples were tested for the presence of PRSV, ToLCV, and CMV using virus-specific polyclonal antibodies (M/s. Leibniz Institute DSMZ—German Collection of Microorganisms and Cell Cultures GmbH, Germany) as per the protocols of Clark and Adams (1977) for double antibody sandwich enzyme-linked immunosorbent assay (DAS-ELISA) and Bhat and Rao (2020) for dot immune-binding assay (DIBA). The samples were extracted in extraction buffer (1 g sample in 10 mL buffer) and centrifuged for 2 min at 12000 rpm at 4 °C. The supernatant was used for the serological detection of the viruses using an automatic ELISA Reader (HER 480 HT Company (Ilford) Ltd., UK).

#### 2.3.2 Molecular detection of the viruses

Molecular detection of the viruses was done by PCR amplification using total DNA in the case of DNA viruses and cDNA in the case of RNA viruses. The specific primers (coat protein-specific) used for the detection of the viruses in the diseased samples are given in Supplementary Table S1.

##### 2.3.2.1 DNA extraction and PCR amplification

The diseased leaf samples (100 mg) were used for total DNA extraction using the DNeasy® Plant Mini extraction kit (Cat. No. 69104, M/s. Qiagen Inc., Germany), following the manufacturer’s Quick-Start protocol. The DNA was finally eluted in 50 μL of DNase-free water and stored in a deep freezer at −20 °C for further use. The yield and quality of the total isolated DNA were assessed using a BioSpectrophotometer (Eppendorf AG, Germany) and confirmed through gel electrophoresis on a 1% agarose gel in 1× Tris–acetate–EDTA (TAE) buffer. The DNA was subjected to PCR amplification to detect the presence of the tomato leaf curl virus using the virus-specific coat protein primers (Supplementary Table S1). The PCR was done in a 25 μL reaction mix containing 12.5 μL master mix (M/s. Takara EmeraldAmp® GT PCR master mix) (Cat. No. RR310A, Japan), 2.5 μL each of forward and reverse primers, 3 μL of template DNA (0.5 μg), and 4.5 μL double-distilled water. The PCR conditions were set with an initial cycle of denaturation at 94 °C for 4 min, followed by 30 cycles of denaturation at 94 °C for 1 min, annealing for 1 min at 50 °C, and extension for 1 min at 72 °C; with a final extension at 72 °C for 10 min in a Thermal Cycler T100^TM^ (BIO-RAD, USA). The PCR products (5 μL each) were resolved in 1.2% agarose gel electrophoresis after staining the gel with ethidium bromide at 2 μL per 50 mL of gel (0.5 μg/mL). The gel after electrophoresis was visualized in a gel documentation system (Gel Doc ™ XR+, M/s. BIO-RAD, USA).

##### 2.3.2.2 RNA extraction and reverse-transcription PCR (RT-PCR)

Total RNA was extracted from the diseased leaf samples using the RPD Trio^TM^ Reagent (M/s. Himedia, Cat No. MB566, India), as per the manufacturer’s protocol. The yield and quality of the total isolated RNAs were assessed using a BioSpectrophotometer (Eppendorf AG, Germany) and confirmed using gel electrophoresis in a 1% agarose gel in RNase-free 1X TAE buffer. RNA was normalized to 1 μg/μL and used as the template for cDNA synthesis. cDNA synthesis of the extracted total RNA was carried out using the Verso cDNA synthesis kit (Cat No. AB-1453A, M/s. Thermo Fisher Scientific Inc., USA) in accordance with the manufacturer’s protocol. The reaction was performed in 20 μL mixture consisting of 4 μL 5X cDNA synthesis buffer, 1 μL each of random primer and RT enhancer, 2 μL dNTP mix, and Verso enzyme mix, 4 μL of PCR-grade water, and 4 μL plant total RNA (0.5 μg). The mix was incubated at 42 °C for 30 min in a thermal cycler for cDNA synthesis and inactivated at 95 °C for 2 min. The cDNA was stored at −20 °C for PCR reactions.

RT-PCR with cDNA was performed using specific primers for detecting the RNA viruses (Supplementary Table S1). The RT-PCR for the detection of PRSV as well as CMV was carried out in 25 μL mixture containing 12.5 μL PCR master mix, 1 μL each of forward and reverse primers, 2.5 μL of template cDNA, and 8.0 μL double-distilled water. The coat protein gene of PRSV was amplified using the following conditions: 4 min at 94 °C; 30 cycles of 1 min at 94 °C, 1 min at 52 °C, and 1 min at 72 °C; with a final elongation at 72 °C for 7 min. The coat protein gene of CMV was amplified by performing a PCR run at 94 °C for 2 min followed by 30 cycles of 1 min at 94 °C, 1 min at 54 °C, and 1 min at 72 °C, with the final elongation at 72 °C for 7 min.

The PCR products (5 μL each) were run on a 1.2 per cent agarose gel in 1X TAE buffer. Gels were stained with ethidium bromide at 2 μL per 50 mL of gel (0.5 μg/mL). The resulting amplicon was documented using a gel documentation system (Gel Doc ™ XR+, M/s. BIO-RAD, USA).

### 2.4 Maintenance of the viruses

Wedge-graft transmission was done to maintain the viruses of the diseased plants. Healthy bitter gourd seedlings (4–5 leaf stage) raised in an insect-proof net house were used as the rootstock. The infected scions were excised from the symptomatic parts (distal vines) of the viruses-infected bitter gourd plants. Wedge grafting was performed for the transmission of viruses. Rootstocks of bitter gourd seedlings were cut 1 cm above the cotyledonary leaves. A vertical slit (1 cm deep) was made, and the infected scion was firmly inserted and properly tied using parafilm. Excess leaves were removed to avoid transpiration loss, and mechanical support to the graft union was provided using a wooden stump. The grafted plants were maintained inside an insect-proof net house to develop the symptoms and maintain the viruses.

### 2.5 Evaluation of *P. indica-*colonized plants against bitter gourd mosaic complex

#### 2.5.1 Pot culture studies

The pot culture studies to evaluate the effect of *P. indica* against the multiple viruses causing bitter gourd mosaic complex were conducted in an insect-proof net house. The virus complex (ToLCV+PRSV+CMV) maintained in the bitter gourd plants, grown in an insect-proof net house, was used as the source of the virus-infected scions.

In pre-inoculation studies, *P. indica*-colonized bitter gourd seedlings were graft-transmitted with the viruses at different intervals. The bitter gourd seedlings were initially raised in pro-trays with sterilized potting mixture as described earlier (2.2). The four leaf-staged seedlings, after removing the debris from the roots with sterile water, were transplanted to pots containing 1 kg of *P. indica*-multiplied potting mixture having 10^6^ cfu g^−1^ of mixture for fungal colonization (Jojy et al., 2020). To the *P. indica*-colonized seedlings, the viruses-infected scions were wedge-grafted at 0, 2, 5, 10, and 15 days after the colonization, as described earlier (2.4). The control seedlings (without *P. indica*) grafted with the virus-infected scion served as a positive control. *Piriformospora indica*-colonized seedlings wedge-grafted with the virus-free scion served as a negative control. The control seedlings wedge-grafted with the virus-free scion (without any treatment) served as an absolute control. The bitter gourd variety chosen was Preethi.

In post-inoculation studies, viruses-infected scions were grafted onto healthy bitter gourd seedlings (4 leaf stage) grown in pots filled with potting mixture; then the viruses-infected seedlings were transplanted to pots filled with 1 kg of *P. indica*-multiplied potting mixture having 10^6^ cfu g^−1^ of mixture for fungal colonization (Jojy et al., 2020) at 0, 2, 5, 10, and 15 days after virus inoculation. The seedlings grafted with viruses-infected scions served as a positive control. *Piriformospora indica*-colonized seedlings wedge-grafted with the virus-free scion served as a negative control. The control seedlings wedge-grafted with the virus-free scion (without any treatment) served as an absolute control. The design of all the pot culture experiments was CRD with 8 treatments, and each treatment was replicated 10 times in each CRD experiment. Five independent CRD experiments were performed separately for pre- and post-inoculation studies. ANOVA of pooled CRD was performed.

#### 2.5.2 Field studies

Field studies were carried out for two seasons, namely, rabi and summer for 3 years between 2022 and 2024 to assess the effect of *P. indica* colonization on the natural incidence and severity of the bitter gourd mosaic complex. A plot of 20 m × 20 m (400 m^2^) was selected and divided into two separate blocks. Pits of 60 cm diameter and 45 cm depth were dug at a spacing of 2 m × 2 m. Fifteen-day-old *P. indica*-colonized and control bitter gourd seedlings, grown as per the procedure described earlier, were transplanted to the pits at a rate of three seedlings per pit for both treatments. The experimental design followed in the field studies was ‘paired *t*-test’ with 20 replications each. The field studies were carried out for the rabi and summer seasons for three consecutive years. One week after transplanting, separate trellises were erected for both blocks, and supporting ropes were also tied for trailing the vines to the trellis.

In pot culture as well as field studies, all cultural operations and fertilizer recommendations for bitter gourd, as per the Package of Practices of Kerala Agricultural University, were followed (KAU, 2016). The number of days taken for the development of symptoms, the nature and type of symptoms, the number of plants infected, and the disease score or grade to assess the severity of the viral disease were recorded. The disease incidence was assessed by the following formula:


$$\text{Disease incidence}\;(\mathrm{DI})=\frac{\text{Number of plants infected}}{\text{Total number of plants observed}}\times 100$$

The disease severity, in terms of vulnerability index (VI), was estimated as per the score or grade chart developed by Chandran et al. (2024). The symptoms were scored on a scale of 0 to 6 as described below:

0 - no symptom
1 - yellow spots on green leaves of normal size
2 - mottling of leaves with dark and light green color
3 - yellowing of leaves with vein banding
4 - blisters and puckering on leaves
5 - distortion of leaves, reduction in leaf size, papery leaves
6 - stunting, rosetting, hairiness, malformed fruits/no fruits

Based on the above-mentioned scale, VI was calculated for each of the treatment.


$$\text{Vulnerability Index}\;(\mathrm{VI})=\frac{\left(\begin{array}{l} 0n_{0}+1n_{1}+2n_{2}\\ +3n_{3}+4n_{4}+5n_{5}+6n_{6} \end{array}\right)}{n_{t}(n_{c}-1)}\times 100$$

Where,

n_0_, n_1_…n_6_—number of plants in categories 0, 1, 2, 3, 4, 5, 6.

n_t_—total number of plants.

n_c_—number of categories.

The presence of CMV, PRSV, and ToLCV was confirmed using serological and molecular methods. Colonization of *P. indica* in bitter gourd roots was also confirmed at different stages of the crop using root staining and microscopic observations mentioned earlier. *Piriformospora indica* colonization at different stages of the crop was also confirmed using Pi-tef primers at the molecular level. Days taken for flowering, percentage fruit set, and average yield were recorded for each treatment.

### 2.6 Biochemical analyses

Leaf samples collected from pot culture experiments at 2, 15, and 30 days after the pre- and post-inoculation studies described in 2.5.1 were subjected to biochemical analyses. *Piriformospora indica*-colonized plants inoculated with viruses by grafting after 15 days of *P. indica* colonization, plants inoculated with viruses by grafting and colonized with *P. indica* after 15 days of virus inoculation, plants (without *P. indica*) grafted with the viruses-infected scion (positive control), *P. indica*-colonized plants grafted with virus-free healthy scion (negative control), and plants grafted with virus-free healthy scion (without any treatment—absolute control) were used to determine ROS accumulation and activities of different antioxidant enzymes.

#### 2.6.1 Detection of reactive oxygen species and hydrogen peroxide

Presence of ROS and H_2_O_2_ was detected using nitro blue tetrazolium (NBT) and 3,3′-diaminobenzidine (DAB) staining, respectively, following the protocols of Kumar et al. (2014) and Matsuo et al. (2015). The sample leaves were collected and washed gently with distilled water to remove extraneous material. The leaves were placed in separate beakers and soaked in NBT (0.2%) or DAB (1 mg/mL) staining solutions. The beakers were fully wrapped immediately with aluminum foil and incubated overnight at room temperature. The stained leaves were soaked in absolute alcohol and kept in a boiling water bath until the leaves turned pale white. Then the leaves were carefully taken and overlaid on a paper towel saturated with 60 per cent glycerol. The areas of leaves where H_2_O_2_ activity was present were visualized as a reddish-brown stain due to the reaction of DAB with the endogenous H_2_O_2_. The activity of ROS was detected as dark blue due to the formation of formazan compound as a result of the reaction of NBT with endogenous ROS.

#### 2.6.2 Estimation of antioxidant enzyme activity

Antioxidant enzymes like catalase (Luck, 1974), superoxide dismutase (Dhindsa et al., 1981), and peroxidase (Srivastava, 1987) were estimated using standard protocols.

To measure the catalase activity, 1 g of sample leaf tissue was ground in 20 mL of 0.0067 M phosphate buffer, pH 7.0, centrifuged for 15 min at 4 °C at 5000 rpm, and the supernatant was taken. An experimental cuvette was filled with 3.0 mL of H_2_O_2_-phosphate buffer, and 40 μL of enzyme extract was added and thoroughly mixed. At 240 nm, the time needed for a 0.05 unit drop in absorbance was measured in a spectrophotometer. The enzyme solution with H_2_O_2_-free phosphate buffer was used as a control. The quantity of enzyme required to reduce the absorbance at 240 nm by 0.05 units was determined as one enzyme unit (EU). The time required for the change of absorbance (Δt) by 0.05 at 240 nm was recorded, and the catalase activity was calculated in enzyme units (EU) per ml of the extract.


$$\text{Catalase activity}\;(\mathrm{EU})=\frac{\mathrm{\Delta A}240}{\Delta_{t}}\times \frac{1}{\text{volume of enzyme extract}\;(\mathrm{mL})}$$

Where ΔA240 is the change in absorbance at 240 nm and Δt is the time interval (in min).

The SOD activity was assessed by its capacity to inhibit the photochemical reduction of NBT. A pre-chilled pestle and mortar (4 °C) was used to homogenize a 1 g leaf sample in 10 mL of ice-cold 50 mM potassium phosphate buffer, pH 7.0, and a pinch of PVP. The homogenate was centrifuged for 10 min at 15,000 g at 4 °C. The resulting supernatant was used as an enzyme extract. The 3 mL reaction mixture included 50 mM phosphate buffer, pH 7.8, 0.1 mM EDTA, 2 μM riboflavin, 75 μM NBT, 13 mM methionine, and 50 μL enzyme extract. After adding riboflavin towards the end, the tubes were shaken and positioned 30 cm below a light source. To trigger the reaction, the light was turned on for 15 min. The light was turned off to halt the reaction, and the tubes were wrapped in aluminum foil or a black cloth. The absorbance was measured in a spectrophotometer at 560 nm. The spectrophotometer was calibrated by setting a blank solution without the addition of the enzyme extract and NBT. A reaction mixture that was kept out of the light did not turn color and was used as the control. NBT was added without the enzyme extract to create an extra reference control. Maximum color development occurred in the reaction mixture lacking enzyme, and this dropped when more enzyme extract was added. According to Giannopolitis and Ries (1977), the volume of enzyme extract utilized in the reaction mixture was plotted against log A560. The volume of enzyme extract that corresponded to 50% inhibition of the reaction was determined from the resulting graph and was regarded as one EU (Beauchamp and Fridovich, 1971).


$$\begin{array}{l} \mathrm{SOD}\;\text{activity}\;(\mathrm{EU})=\frac{\left(A_{\text{control}}-A_{\text{sample}}\right)}{A_{\text{control}}}\\ \times \frac{100}{\text{fresh weight of leaf}\;(g)}\times \frac{1}{50} \end{array}$$

Where A_control_ is the absorbance of control at 560 nm (without the enzyme extract) and A_sample_ is the absorbance of the reaction mixture with enzyme extract at 560 nm.

To determine the activity of peroxidase (PO), a 1 g leaf sample was homogenized in a pre-chilled pestle and mortar in 5 mL of sodium phosphate buffer, pH 6.5, and a pinch of polyvinyl pyrrolidone. After passing the homogenate through muslin cloth, the filtrate was centrifuged at 4 °C for 15 min at 5000 rpm. To estimate PO activity, the supernatant was used as the enzyme extract. Three mL of 0.05 M pyrogallol and 50 μL of enzyme extract were taken out of the sample, and reference cuvettes made up the reaction mixture. At 420 nm, the mixture was placed in a spectrophotometer, and the reading was set to zero. After adding 1 mL of 1% H_2_O_2_ to the sample cuvettes, the reaction was started. For 180 s, the absorbance readings (at 420 nm) were monitored every 30 s. PO activity was measured as changes in absorbance per min per g of tissue on a fresh weight basis. A unit of peroxidase (EU) is recorded as the change in absorbance per min at 420 nm.


$$\text{Peroxidase activity}\;(\mathrm{EU})=\frac{\Delta A_{420}}{\Delta_{t}}\times \frac{1}{\text{volume of enzyme extract}\;(\mathrm{ml})}$$

Where ΔA_420_ is the change in absorbance at 420 nm and Δt is the time interval (in min).

### 2.7 RNA extraction and RT-qPCR of different ROS marker and antioxidant genes

RNA was isolated from the leaf samples, and RT-PCR was performed. The reverse transcription of extracted total RNA was carried out using the Verso cDNA synthesis kit (Cat No. AB-1453A, M/s. Thermo Fisher Scientific Inc., USA) in accordance with the manufacturer’s protocol. RT-qPCR was performed as described by Li et al. (2019). The relative expression of different ROS marker genes and antioxidant genes was studied in the best treatments from the pot culture studies with the respective infected samples and control. The relative expression of ROS marker genes (*WRKY40, MYB51, CML37*) and H_2_O_2_ marker genes (*AGP5*) in the nucleus, ROS marker gene (*LOX2*) and H_2_O_2_ marker gene (*PTOX*) in the chloroplast, and ROS marker genes (*HSPRO1, DIC2*) and H_2_O_2_ marker gene (*PRX*) in the mitochondria were quantified. Similarly, the relative expression of antioxidant genes localized in the nucleus (*CSD1, APX1*), chloroplast (*FSD1, FSD2*), and mitochondria (*MSD1, AOX2*) was also determined. The reference gene, *GAPDH*, was used to normalize the cDNA concentration for each sample. The relative expressions of genes were determined using the formula of Pfaffl (2001). Details of gene-specific primers used in the study are given in Supplementary Table S2.

### 2.8 Statistical analysis

All the *in vitro* experiments and pot culture studies followed CRD with 5 independent experiments. The data were pooled and analyzed using one-way analysis of variance (ANOVA). Statistical significance between the treatments was compared using the least significant difference (LSD) test at the *p* < 0.05 probability level. Field experiments were laid out as per paired *t*-test design and performed in both rabi and summer seasons over 3 consecutive years. All the statistical analyses were performed using the statistical software of Kerala Agricultural University, GRAPES 1.0.0, developed by Gopinath et al. (2020).

## 3 Results

### 3.1 *Piriformospora indica* colonization in the roots of bitter gourd plants

Within 2 weeks, *P. indica* has fully developed in 9 cm diameter Petri plates with PDA medium (Supplementary Figure 1A). Similarly, the fungal mat was formed within 14 days of inoculation in the PDB medium. Initially, the fungal disc grew as a cotton ball-like mycelial growth in the broth and remained at the base of the flask (Supplementary Figure 1B). At later stages of growth, a thick fungal mat in layers was formed above the broth with a slight submergence. When the fungus was mass-multiplied, visible mycelial growth appeared in the coco peat-farm yard manure mix from 5 days post-inoculation (Supplementary Figure 1C). The complete mycelial run took around 7 days at room temperature. The bitter gourd seedlings were raised in *P. indica*-multiplied potting mixture, and the roots were tested for the presence of *P. indica* using a staining technique. Fungal structures (mycelia) were visible in the roots from the 3^rd^ day after germination (DAG; Supplementary Figure 2A). Nonetheless, chlamydospore formation in bitter gourd roots was observed from 10 DAG (Supplementary Figure 2B) and was clearly visible by 15 DAG in the roots (Supplementary Figure 2C). The effect of endophyte colonization on the growth and vigor of bitter gourd seedlings was visible right from germination, and significant changes were seen from 5 DAG (Supplementary Figure 3).

### 3.2 Bitter gourd mosaic complex disease manifested a wide range of symptoms

**Figure 1:**
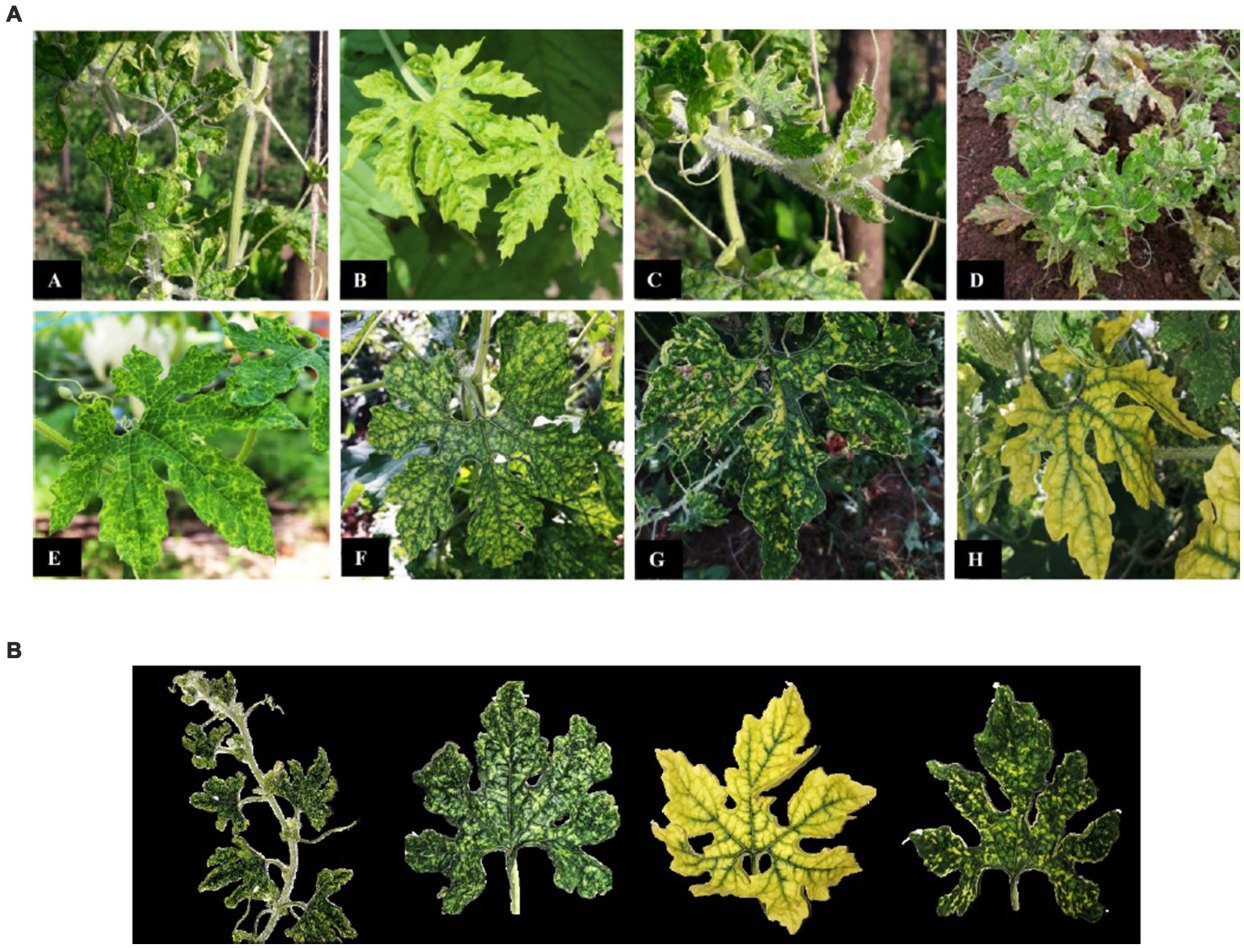
**(A)** General symptoms of bitter gourd mosaic complex disease observed in the fields. (A) Upward leaf curling, (B) mosaic, (C) stunting and hairiness, (D) rosetting, (E) vein clearing, (F) vein banding, (G) leaf mottling, blistering, and distortion, (H) yellowing of leaves with downward curling. **(B)** Specific symptoms produced by ToLCV (A: Upward leaf curling, mosaic and hairiness), PRSV (B: Blisters and puckering on leaves; and C: Yellowing of leaves with downward curling), and CMV (D: Mottling and distortion of leaves in bitter gourd plants).

The diseased samples collected from bitter gourd fields showed different types of symptoms. General as well as specific symptoms of the viral disease were observed. The most common symptoms observed in the diseased bitter gourd plants were mosaic, stunted growth, yellowing of leaves with downward curling, blistering, puckering of leaves, upward leaf curling, chlorosis, yellow spots on leaves, and hairiness. Other symptoms included rosetting, mottling of leaves, leaf distortion, vein banding, and vein clearing (Figure 1A). Specific symptoms were found to be associated with certain viruses. It was found that upward leaf curling and hairiness were common symptoms in ToLCV-infected plants, whereas yellowing of leaves with downward curling, blistering, and puckering on leaves were found to be associated with PRSV infection. Similarly, mottling and distortion of leaves were observed specifically in plants tested positive for CMV (Figure 1B; Supplementary Table S3). Combined infection of ToLCV and PRSV resulted in upward curling, blistering, and puckering of leaves; whereas the infection of ToLCV and CMV resulted in upward curling, mottling, and distortion of leaves. Interestingly, PRSV and CMV infection led to downward curling, puckering, and yellowing of leaves with or without distortion. Combined infection of the three viruses resulted in upward curling, blistering, puckering and distortion of leaves, hairiness of leaves and stem, and stunted growth (Supplementary Table S3). More often, ToLCV, PRSV, and CMV together resulted in severe stunting of plants, leading to complete cessation of flowering, resulting in 100% crop loss.

### 3.3 ToLCV, PRSV, and CMV were detected in bitter gourd mosaic complex-affected leaves through serological and molecular methods

**Figure 2:**
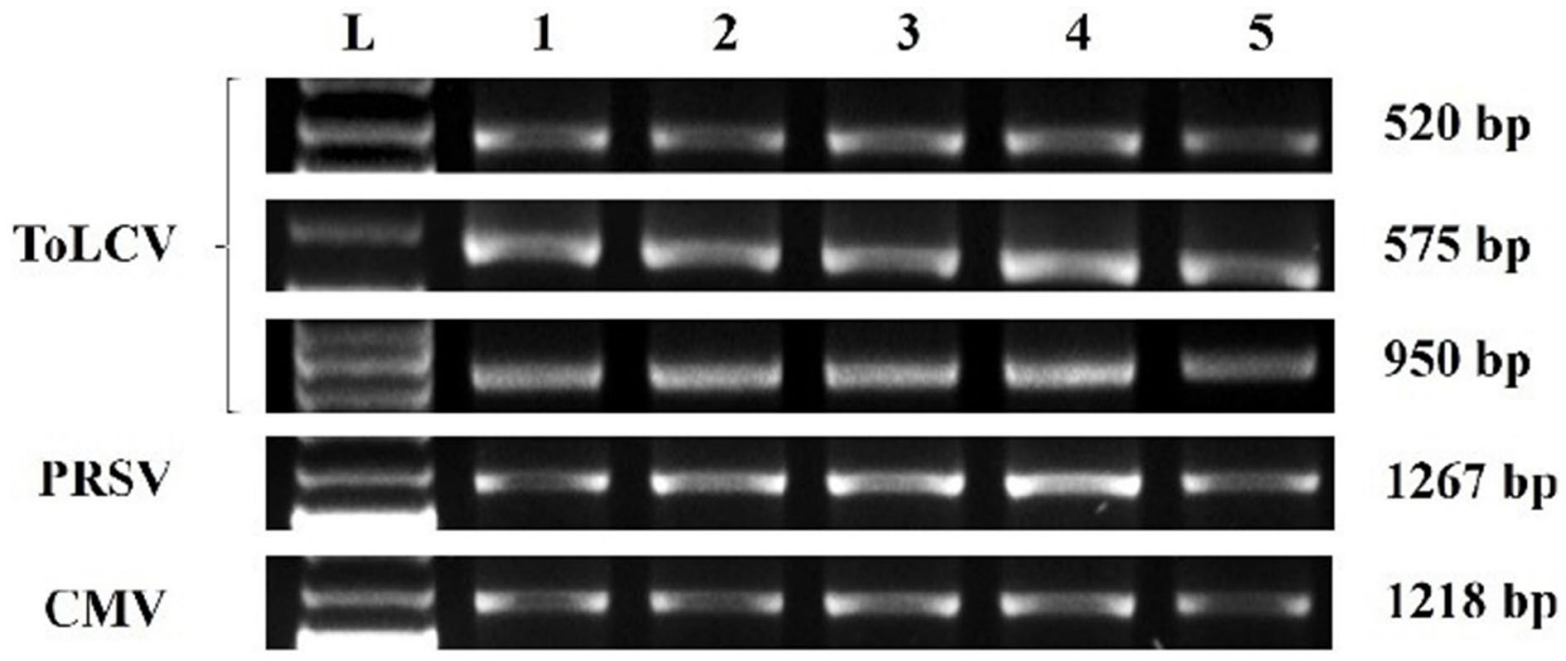
Electrophoresis gel image of amplified DNA of ToLCV and amplified cDNA of PRSV and CMV using respective coat protein specific primers in bitter gourd leaf samples showing symptoms of bitter gourd mosaic complex disease. Lane L—1 kb DNA ladder, Lanes 1–5: bitter gourd leaf samples showing symptoms of bitter gourd mosaic complex disease.

The presence of ToLCV, PRSV, and CMV in the BGMC diseased leaf samples was confirmed through DAS-ELISA and DIBA using the virus-specific polyclonal antibodies purchased from Leibniz Institute DSMZ-German Collection of Microorganisms and Cell Cultures GmbH, Germany. In DAS-ELISA, BGMC diseased leaf samples showed nearly a 10-fold increase in the OD values at 405 nm with the three virus-specific polyclonal antibodies compared to the healthy control samples (Supplementary Table S4). Similarly, with DIBA, BGMC affected leaf samples showed nearly a 4–5-fold increase in mean intensity value with the virus-specific polyclonal antibodies on the nitrocellulose membrane (Supplementary Table S4). These serological results clearly indicate that the BGMC affected samples had a combined infection of all three viruses: ToLCV, PRSV, and CMV. As expected, the coat protein primer of ToLCV yielded an amplicon of 950 bp, confirming the presence of ToLCV in BGMC. Similarly, the coat protein primers of PRSV and CMV yielded the expected amplicons of 1,267 bp and 1,218 bp, respectively, confirming the association of PRSV and CMV with BGMC at the molecular level (Figure 2). Serological and molecular studies clearly demonstrated the association of ToLCV, PRSV, and CMV with BGMC disease in Kerala, India.

### 3.4 *Piriformospora indica* colonization significantly inhibited the symptoms of bitter gourd mosaic complex disease produced by combined infection of ToLCV, PRSV, and CMV

The triple virus complex (ToLCV, PRSV, CMV) maintained in bitter gourd plants in the insect-proof net house was used as the source of viruses in the pot culture studies. In pre-inoculation studies, the symptoms of BGMC appeared earlier in the control plants (7.20 days) compared to *P. indica*-colonized plants. Plants colonized with *P. indica* and inoculated with viruses on the same day took 12.10 days for the appearance of the first symptoms, whereas colonized plants inoculated with the viruses by grafting at 15 days after colonization (DAC) showed the first symptoms only by 14.30 days (Table 1). Thus, *P. indica* colonization significantly delayed the appearance of symptoms of BGMC disease compared to the infected controls. Moreover, early colonization of *P. indica* further delayed symptom expression.

**Table 1:** Effect of *P. indica* colonization in bitter gourd plants on days taken for symptom development, incidence, and severity of bitter gourd mosaic complex following artificial inoculation of ToLCV, PRSV, and CMV in the pre-inoculation studies.

Treatments	Days taken for symptom appearance^*^	Disease incidence (%)			Disease severity as vulnerability index		
		15 DAI	45 DAI	75 DAI	15 DAI	45 DAI	75 DAI
+Pi/+V_0_	12.10^d^ ± 0.56	100 ± 0.00	90.0^e^ ± 7.07	64.0^c^ ± 5.44	47.43^c^ ± 2.79	34.07^d^ ± 2.44	32.06^d^ ± 1.82
+Pi/+V_2_	12.80^c^ ± 0.63	100 ± 0.00	78.0^d^ ± 4.40	46.0^b^ ± 8.04	36.41^b^ ± 1.82	32.73^d^ ± 2.79	25.38^c^ ± 3.21
+Pi/+V_5_	13.30^b^ ± 0.48	98.0^c^ ± 4.32	46.0^c^ ± 5.47	40.0^b^ ± 5.04	34.74^b^ ± 2.98	27.05^c^ ± 5.83	19.04^b^ ± 1.49
+Pi/+V_10_	14.00^a^ ± 0.47	62.0^b^ ± 4.47	28.0^b^ ± 4.12	22.0^a^ ± 4.14	25.38^a^ ± 4.48	19.37^b^ ± 1.49	12.02^a^ ± 1.82
+Pi/+V_15_	14.30^a^ ± 0.48	44.0^a^ ± 5.47	18.0^a^ ± 4.28	16.0 ^a^ ± 5.32	21.38^a^ ± 4.16	14.69^a^ ± 1.82	9.02^a^ ± 2.24
+V alone	7.20^e^ ± 0.63	100 ± 0.00	100 ± 0.00	100 ± 0.00	77.49 ± 1.90	78.16 ± 4.48	84.17 ± 2.79
+Pi alone	–	0	0	0	0	0	0
Absolute Control	–	0	0	0	0	0	0
SE (m)	0.174	1.528	2.160	2.517	1.443	1.584	1.034
CD (0.05)	0.493	4.459	6.305	7.345	4.212	4.624	3.018

*Piriformospora indica*-colonized bitter gourd seedlings (4 leaf stage) were graft-transmitted with scions having ToLCV, PRSV, and CMV at 0 (+Pi/+V_0_), 2 (+Pi/+V_2_), 5 (+Pi/+V_5_), 10 (+Pi/+V_10_), and 15 (+Pi/+V_15_) days after the colonization, as described in the Materials and Methods. The control seedlings (without *P. indica*) wedge-grafted with the virus-infected scion was positive control (+V alone), while *P. indica*-colonized seedlings wedge-grafted with virus-free scion served as negative control (+Pi alone). The seedlings wedge-grafted with the virus-free scion (without any treatment) served as the absolute control. ^*^Values are the mean of 50 replications ± standard deviation. Ten plants per treatment per experiment. Five independent experiments were conducted. Superscripts with same letters indicate on-par values, and those with different letters indicate a significant difference at the 5 per cent level of significance. DAI: days after inoculation of the virus by grafting. Pooled CRD. SE, standard error; CD, critical difference at the 5% level of significance.

**Figure 3:**
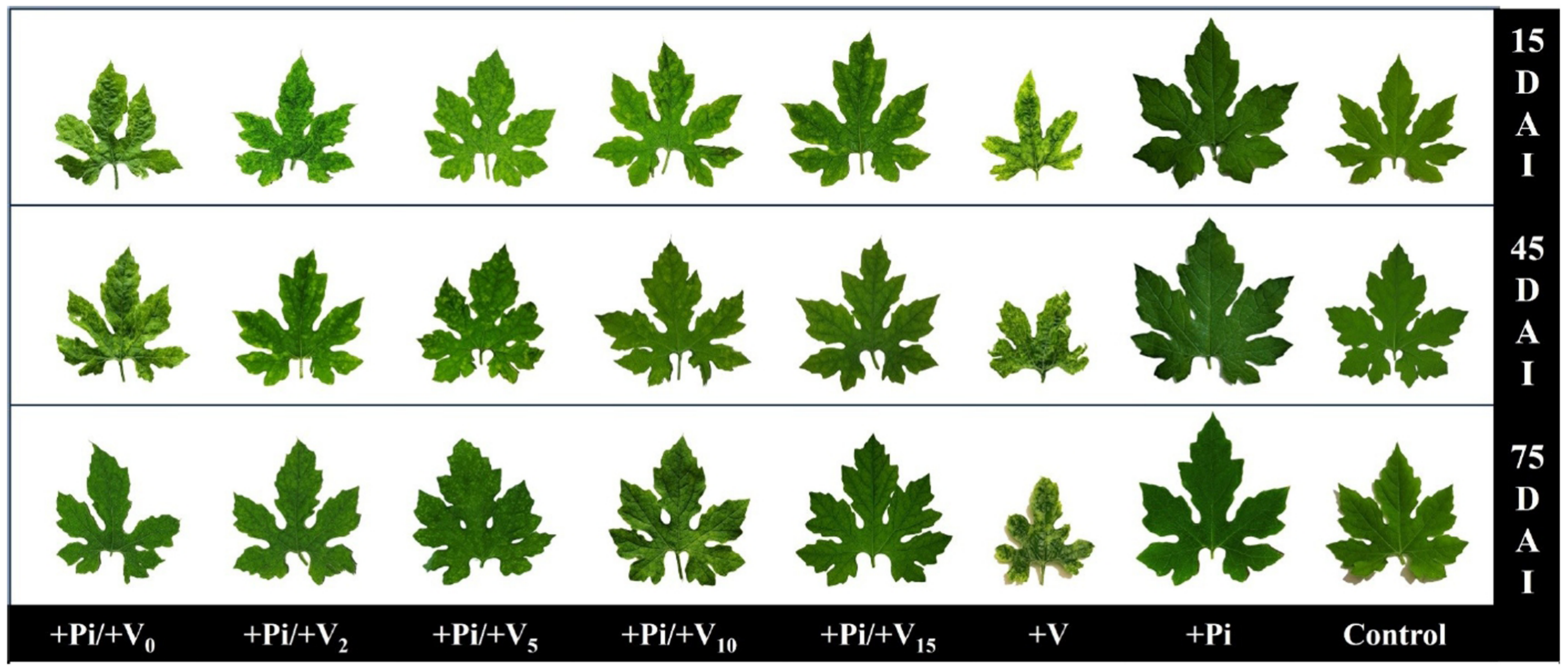
Effect of *P. indica*-colonization on symptom development of bitter gourd mosaic complex incited by ToLCV, PRSV, and CMV in bitter gourd leaves in the pre-inoculation studies at 15 DAI, 45 DAI, and 75 DAI. *P. indica*-colonized bitter gourd seedlings (4 leaf-stage) were graft-transmitted with scions having ToLCV, PRSV, and CMV at 0 (+Pi/+V0), 2 (+Pi/+V2), 5 (+Pi/+V5), 10 (+Pi/+V10), and 15 (+Pi/+V15) days after the colonization as described in the materials and methods. The control seedlings (without *P. indica*) wedge-grafted with the viruses-infected scion was positive control (+V), and *P. indica*-colonized seedlings wedge grafted with viruses-free scion served as negative control (+Pi). The seedlings wedge grafted with viruses-free scion (without any treatment) served as absolute control. Ten plants per treatment. Representative picture of five independent experiments are shown. DAI: days after inoculation of the viruses by grafting.

The infected control plants showed symptoms such as mosaic, upward leaf curling, yellowing of leaves with downward curling, yellow spots on leaves, leaf distortion, stunting, increased hairiness, and severe fruit malformation (Table 2). In contrast, fewer symptoms appeared in *P. indica*-colonized plants inoculated with the viruses by wedge grafting. All of the symptoms except distortion and yellowing of leaves with downward curling appeared in the *P. indica*-colonized plants inoculated with the viruses on the same day of colonization, 2 DAC, and 5 DAC. On the contrary, mild symptoms, such as yellow spots on leaves and hairiness, appeared in the *P. indica*-colonized plants inoculated with the viruses on 10 DAC and 15 DAC. The yellow spots on leaves in these plants faded at later stages of plant growth, and the plants appeared nearly similar to healthy ones (Figure 3).

**Table 2:** Effect of *P. indica*-root colonization in bitter gourd plants on the type of symptoms of bitter gourd mosaic complex following artificial inoculation of ToLCV, PRSV, and CMV in the pre-inoculation studies at 75 DAI.

Type of symptoms of BGMC	Treatments							
	+Pi/+V_0_	+Pi/+V_2_	+Pi/+V_5_	+Pi/+V_10_	+Pi/+V_15_	+V alone	+Pi alone	Absolute Control
Upward leaf curling	++	++	+	−	−	++++	−	−
Mosaic	++	++	+	−	−	++++	−	−
Yellowing of leaves with downward curling	+	+	−	−	−	++++	−	−
Yellow spots in leaves	++	++	+	+	+	++++	−	−
Hairiness	++	++	+	+	+	++++	−	−
Stunted growth	++	++	−	−	−	++++	−	−
Distortion of leaves	++	++	+	−	−	++++	−	−
Fruit malformation	+	+	−	−	−	++++	−	−

*Piriformospora indica*-colonized bitter gourd seedlings (4 leaf stage) were graft-transmitted with scions having ToLCV, PRSV, and CMV at 0 (+Pi/+V_0_), 2 (+Pi/+V_2_), 5 (+Pi/+V_5_), 10 (+Pi/+V_10_), and 15 (+Pi/+V_15_) days after colonization, as described in the Materials and Methods. The control seedlings (without *P. indica*) wedge-grafted with the virus-infected scion served as the positive control (+V alone), and *P. indica*-colonized seedlings wedge-grafted with virus-free scion served as negative control (+Pi alone). The seedlings wedge-grafted with the virus-free scion (without any treatment) served as the absolute control. Ten plants per treatment per experiment were used. Five independent experiments were conducted. ++++ Severe, +++ moderately severe, ++ moderate, + mild, − symptoms absent, from 50 replications per treatment.

BGMC disease incidence and severity of the viral disease also differed among the colonized and control plants. Severe disease incidence was recorded in all the virus-inoculated bitter gourd plants at 15 DAI, whereas mild to moderate forms of disease incidence were observed in the *P. indica*-colonized plants inoculated with the viruses on the same day, 2 DAC, and 5 DAC. Interestingly, the disease incidence was reduced to 40% at 45 DAI and 75 DAI (Table 1). *Piriformospora indica*-colonized plants inoculated with the viruses at 10 DAC and 15 DAC significantly reduced BGMC disease incidence to 22 and 16%, respectively (Table 1). However, at later stages, there were no visible symptoms of viral disease in new flushes in these treatments; hence, the incidence was recorded as zero. The severity of the viral disease was also compared based on the vulnerability index in different treatments (Table 1; Figure 3). It was observed that the VI increased from 77.49 in the infected plants at 15 DAI to 84.17 at 75 DAI. This trend was reversed in the case of *P. indica*-colonized plants inoculated with the viruses at different intervals after colonization. These plants showed a substantial decrease in the severity of viral disease from 15 DAI to 75 DAI. Prominent results were observed in the case of *P. indica*-colonized plants inoculated with the viruses at 15 DAC; whereas the VI reduced from 21.38 at 15 DAI to 14.69 at 45 DAI, which in turn was further reduced to 9.02 at 75 DAI (Table 1).

**Figure 4:**
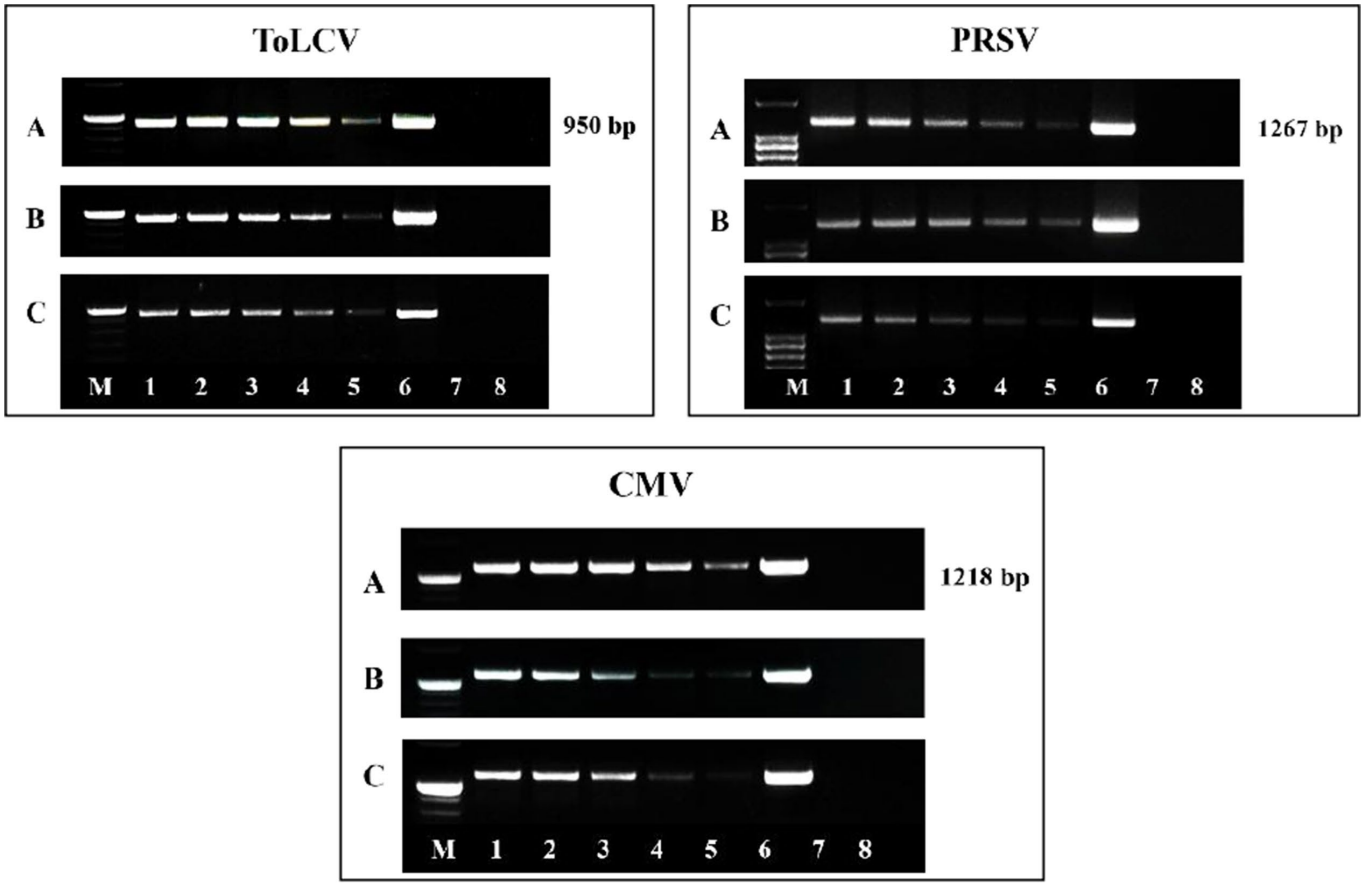
Electrophoresis gel image of amplified DNA of ToLCV and amplified cDNA of PRSV and CMV using their respective coat protein specific primers at **(A)** 15 DAI, **(B)** 45 DAI, and **(C)** 75 DAI from the pre-inoculation studies. Lanes: M: 1 kb DNA ladder; 1–8: 8 treatments from the pre- inoculation studies. *Piriformospora indica*-colonized bitter gourd seedlings (4 leaf-stage) were graft-transmitted with scions having ToLCV, PRSV, and CMV at 0 (1: +Pi/+V0), 2 (2: +Pi/+V2), 5 (3: +Pi/+V5), 10 (4: +Pi/+V10) and 15 (5: +Pi/+V15) days after the colonization as described in the materials and methods. The control seedlings (without *P. indica*) wedge-grafted with the viruses-infected scion was positive control (6: +V alone) and *P. indica*-colonized seedlings wedge grafted with viruses-free scion served as negative control (7: +Pi alone). The seedlings wedge grafted with viruses-free scion (without any treatment) served as absolute control (8). Representative picture of five independent experiments were done. DAI: days after inoculation of virus by grafting.

At different stages, the presence of three viruses, such as ToLCV, PRSV, and CMV, was detected, and the viruses’ titers were assessed both by DAS-ELISA (Supplementary Table S5; Supplementary Figure 4) as well as PCR methods (Figure 4). The presence of all three viruses was detected in all the plants graft-inoculated with the viruses. The titers of all three viruses were high in the inoculated control plants; whereas the titers showed a gradual reduction in the *P. indica*-colonized plants inoculated with the viruses. *Piriformospora indica*-colonized plants inoculated with viruses recorded the lowest viral titer at 75 DAI (Supplementary Table S5; Supplementary Figure 4; Figure 4). These results clearly demonstrated that *P. indica* could drastically reduce the BGMC symptoms produced by the combined infection of ToLCV, PRSV, and CMV by inhibiting their replication. To the best of our knowledge, this is the first scientific evidence of the ability of *P. indica* to simultaneously inhibit multiple viruses when used prophylactically.

**Figure 5:**
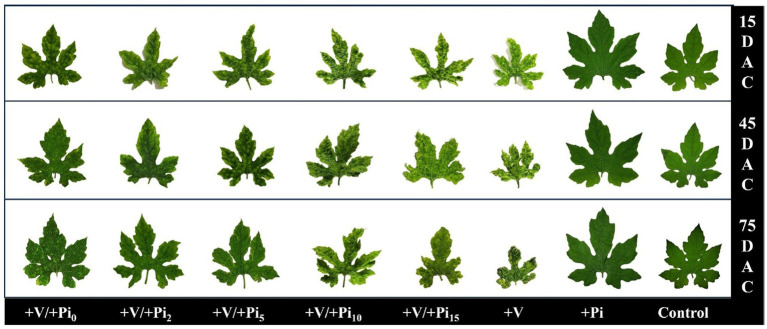
Effect of *P. indica*-colonization on symptom development of bitter gourd mosaic complex incited by ToLCV, PRSV, and CMV in bitter gourd leaves in the post-inoculation studies at 15 DAC, 45 DAC, and 75 DAC. The viruses-infected scions (ToLCV, PRSV, and CMV) were graft-transmitted to 4 leaf-stage healthy bitter gourd seedlings grown in pots filled with potting mixture; and then the viruses- inoculated-seedlings were transplanted to pots filled with *P. indica*-multiplied potting mixture having 10^6^ cfu g^-1^ for the fungal colonization at 0 (+V/+Pi_0_), 2 (+V/+Pi_2_), 5 (+V/+Pi_5_), 10 (+V/+Pi_10_), and 15 (+V/+Pi_15_) days after the viruses inoculation as described in the materials and methods. The control seedlings (without *P. indica*) wedge-grafted with the viruses-infected scion was positive control (+V) and *P. indica*- colonized seedlings wedge grafted with viruses-free scion as negative control (+Pi). The seedlings wedge grafted with viruses-free scion (without any treatment) served as absolute control. Ten plants per treatment. Representative picture of five independent experiments are shown. DAC: days after colonization of *P. indica*.

**Figure 6:**
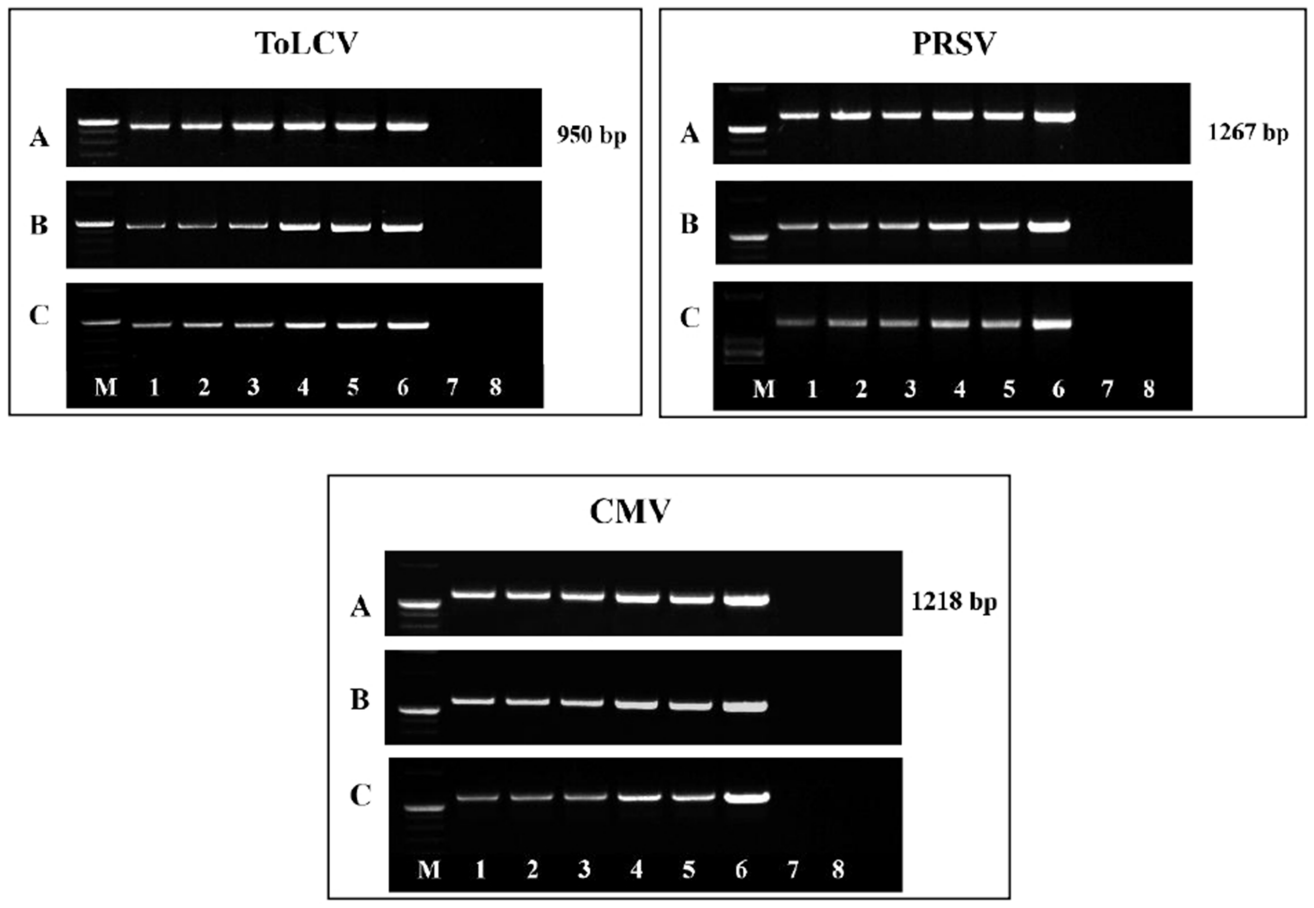
Electrophoresis gel image of amplified DNA of ToLCV and amplified cDNA of PRSV and CMV using their respective coat protein specific primers at **(A)** 15 DAC, **(B)** 45 DAC, and **(C)** 75 DAC from the post-inoculation studies. Lanes: M: 1 kb DNA ladder; 1–8: 8 treatments from the post- inoculation studies. The viruses-infected scions (ToLCV, PRSV and CMV) were graft-transmitted to 4 leaf-stage healthy bitter gourd seedlings grown in pots filled with potting mixture; and then the viruses-inoculated-seedlings were transplanted to pots filled with *P. indica*-multiplied potting mixture having 10^6^ cfu g^-1^ for the fungal colonization at 0 (1: +V+Pi_0_), 2 (2: +V+Pi_2_), 5 (3: +V+Pi_5_), 10 (4: +V+Pi_10_) and 15 (5: +V+Pi_15_) days after the viruses inoculation as described in the materials and methods. The control seedlings (without *P. indica*) wedge-grafted with the viruses-infected scion were positive control (6: +V alone) and *P. indica*-colonized seedlings wedge grafted with viruses- free scion served as negative control (7: +Pi alone). The seedlings wedge grafted with viruses-free scion (without any treatment) served as absolute control (8). Representative picture independent experiments are shown. DAC, days after colonization of *P. indica*.

We further extended the research to determine the curative effect of *P. indica* in managing BGMC. In post-inoculation studies, bitter gourd plants were inoculated with the three viruses, and *P. indica* was introduced into the virus-inoculated plants at different intervals. Similar to pre-inoculation studies, the symptoms of virus infection appeared earlier in the control plants compared to *P. indica*-colonized plants (Table 3; Figure 5). In the inoculated plants, the symptoms appeared within 7.70 days compared to *P. indica*-colonized plants. Plants inoculated by grafting and then colonized with *P. indica* on the same day exhibited symptoms by 12.50 days. Symptoms appeared in the virus-inoculated plants by 12.10 and 11.10 days when colonized with *P. indica* after 2 DAI and 5 DAI, respectively (Table 3). The disease incidence was 100 percent in the inoculated plants without *P. indica*. However, BGMC incidence was marginally reduced in the inoculated plants colonized with *P. indica* within 5 days (Table 3) and was more pronounced at 45 DAC and 75 DAC. Though the disease incidence was higher, there was a significant reduction in the severity of symptoms in the *P. indica*-colonized plants, especially at 45 DAC and 75 DAC, compared to the infected controls (Table 4; Figure 5). These results were further validated by assessing the titer of ToLCV, PRSV, and CMV by serological as well as molecular methods at 15 DAC, 45 DAC, and 75 DAC (Supplementary Table S6; Supplementary Figure 5; Figure 6). The post-colonization of *P. indica* in the infected plants also resulted in the gradual reduction of the titer of all three viruses. Notable reduction in the titer of the viruses was observed in the case of the inoculated plants colonized with *P. indica* on the same day, 2 DAI, and 5 DAI, which also points to the curative effect of *P. indica* in managing BGMC.

**Table 3:** Effect of *P. indica* colonization in bitter gourd plants on days taken for symptom development, incidence, and severity of bitter gourd mosaic complex following artificial inoculation of ToLCV, PRSV, and CMV in the post-inoculation studies.

Treatments	Days taken for symptom appearance^*^	Disease incidence (%)			Disease severity as vulnerability index		
		15 DAI	45 DAI	75 DAI	15 DAI	45 DAI	75 DAI
+V/+Pi_0_	12.50^a^ ± 1.26	100 ± 0.00	78.0^a^ ± 4.07	62.0^a^ ± 4.32	46.43^a^ ± 2.47	37.41^a^ ± 4.35	33.07^a^ ± 1.82
+V/+Pi_2_	12.10^b^ ± 0.99	100 ± 0.00	82.0^ab^ ± 4.28	76.0^b^ ± 5.48	51.10^b^ ± 2.24	45.42^b^ ± 1.39	37.07^a^ ± 2.47
+V/+Pi_5_	11.10^c^ ± 0.99	100 ± 0.00	88.0^b^ ± 4.47	86.0^c^ ± 5.24	60.79^c^ ± 1.90	49.77^c^ ± 2.17	44.42^b^ ± 2.79
+V/+Pi_10_	8.10^d^ ± 0.87	100 ± 0.00	100 ± 0.00	100 ± 0.00	65.46^d^ ± 2.17	55.44^d^ ± 1.82	48.43^b^ ± 2.36
+V/+Pi_15_	7.90^d^ ± 0.73	100 ± 0.00	100 ± 0.00	100 ± 0.00	74.15^e^ ± 2.79	61.46^e^ ± 2.98	53.44^c^ ± 7.37
+V alone	7.70^d^ ± 0.48	100 ± 0.00	100 ± 0.00	100 ± 0.00	76.49 ± 0.74	79.49 ± 2.79	85.50 ± 1.82
+Pi alone	–	0	0	0	0	0	0
Absolute control	–	0	0	0	0	0	0
SE (m)	0.292	0	1.414	1.633	0.964	1.235	1.639
CD (0.05)	0.829	0	4.128	4.766	2.814	3.604	4.784

The virus-infected scions (ToLCV, PRSV, and CMV) were graft-transmitted to four leaf-staged healthy bitter gourd seedlings grown in pots filled with potting mixture. The virus-inoculated seedlings were then transplanted to pots filled with *P. indica*-multiplied potting mixture having 10^6^ cfu g^−1^ for fungal colonization at 0 (+V/+Pi_0_), 2 (+V/+Pi_2_), 5 (+V/+Pi_5_), 10 (+V/+Pi_10_) and 15 (+V/+Pi_15_) days after virus inoculation, as described in the Materials and Methods. The control seedlings (without *P. indica*) wedge-grafted with the virus-infected scion served as the positive control (+V alone), while *P. indica*-colonized seedlings wedge-grafted with virus-free scion served as negative control (+Pi alone). The seedlings wedge-grafted with the virus-free scion (without any treatment) served as the absolute control. ^*^Values are the mean of 50 replications ± standard deviation. Ten plants per treatment per experiment. Five independent experiments were conducted. Superscripts with same letters indicate on-par values, and those with different letters indicate a significant difference at the 5 percent level of significance. DAI: days after inoculation of the virus by grafting. Pooled CRD. SE: standard error. CD: critical difference at the 5% level of significance.

**Table 4:** Effect of *P. indica*-root colonization in bitter gourd plants on the type of symptoms of bitter gourd mosaic complex following artificial inoculation of ToLCV, PRSV, and CMV in the post-inoculation studies at 75 DAI.

Symptoms	Treatments							
	+V + Pi_0_	+V + Pi_2_	+V + Pi_5_	+V + Pi_10_	+V + Pi_15_	+V alone	+Pi alone	Absolute control
Upward leaf curling	−	+	+	++	++	++++	−	−
Mosaic	−	+	+	++	++	++++	−	−
Yellowing of leaves with downward curling	−	−	+	+	+	++++	−	−
Yellow spots in leaves	+	+	++	++	+++	++++	−	−
Hairiness	+	+	++	++	+++	++++	−	−
Stunted growth	−	−	+	++	++	++++	−	−
Distortion of leaves	−	−	+	++	++	++++	−	−
Fruit malformation	−	−	+	+	+	++++	−	−

The virus-infected scions (ToLCV, PRSV, and CMV) were graft-transmitted to four leaf-staged healthy bitter gourd seedlings grown in pots filled with potting mixture. The virus-inoculated seedlings were then transplanted to pots filled with *P. indica*-multiplied potting mixture having 10^6^ cfu g^−1^ for fungal colonization at 0 (+V+Pi_0_), 2 (+V+Pi_2_), 5 (+V+Pi_5_), 10 (+V+Pi_10_), and 15 (+V+Pi_15_) days after virus inoculation, as described in the Materials and Methods. The control seedlings (without *P. indica*) wedge-grafted with the virus-infected scion served as the positive control (+V alone), while *P. indica*-colonized seedlings wedge-grafted with virus-free scion served as negative control (+Pi alone). The seedlings wedge-grafted with the virus-free scion (without any treatment) served as the absolute control. Ten plants per treatment per experiment were used. Five independent experiments were conducted. ++++ Severe, +++ moderately severe, ++ moderate, + mild, − symptoms absent, from 50 replications per treatment.

### 3.5 *Piriformospora indica* diminished the reduction of growth and yield of bitter gourd plants affected by bitter gourd mosaic complex disease

The biometric observations such as the number of days taken for flowering, percent fruit set, and yield were recorded from both pre- and post-inoculation studies. In the pre-inoculation studies, the number of days taken for flowering was substantially less, and fruit set and yield were significantly higher in the *P. indica*-colonized plants that were not inoculated with the viruses (Table 5). The absolute control plants took 34.60 days for flowering, whereas the *P. indica* colonized plants started flowering by 25.20 days after transplanting (DAT). On the contrary, the ToLCV, PRSV, and CMV-infected control plants took 43.80 days for flowering. Surprisingly, the *P. indica*-colonized plants with the viruses at 10 DAC and 15 DAC took only 26.10 and 25.80 days, respectively, for flowering, which was comparable with the *P. indica*-colonized plants (Table 5). Hence, it is inferred that *P. indica* colonization has induced early flowering even in the virus-inoculated plants compared to the absolute control and the virus-alone infected plants. Again, the fruit setting was significantly higher in the *P. indica*-colonized plants inoculated with the viruses at 10 DAC and 15 DAC, with 42.07 and 48.46%, respectively, compared to a mere 6.42% in the virus-inoculated plants. It is noteworthy that the healthy control and *P. indica*-colonized plants had higher fruit sets of 54.90 and 62.57%, respectively (Table 5). Similarly, *P. indica*-colonized plants inoculated with the viruses at 10 DAC and 15 DAC produced 2.01 and 2.32 kg plant^−1^, respectively, compared to the lowest fruit yield of 0.07 kg plant^−1^ in the virus-alone infected plants. *Piriformospora indica*-colonized plants not inoculated with the viruses produced the highest yield of 2.72 kg plant^−1^ (Table 5). These results clearly demonstrate that *P. indica* could diminish the loss of growth and yield due to the multiple virus infections of ToLCV, PRSV, and CMV in bitter gourd plants.

**Table 5:** Effect of *P. indica* colonization in bitter gourd plants on yield parameters in the pre-inoculation and post-inoculation studies on artificial inoculation of ToLCV, PRSV, and CMV inciting bitter gourd mosaic complex disease.

Pre-inoculation studies^*^				Post-inoculation studies^*^			
Treatments	Days taken for flowering	Fruit setting (%)	Average yield (kg plant^−1^)	Treatments	Days taken for flowering	Fruit setting (%)	Average yield (kg plant^−1^)
+Pi+V_0_	36.20^a^ ± 0.42	33.75^d^ ± 12.50	0.17^fg^ ± 0.05	+V + Pi_0_	36.5^d^ ± 0.85	31.91^c^ ± 10.10	0.171^c^ ± 0.023
+Pi+V_2_	36.00^a^ ± 0.47	35.73^d^ ± 10.67	0.24^ef^ ± 0.04	+V + Pi_2_	38.0^c^ ± 0.47	19.16^d^ ± 7.45	0.124^cd^ ± 0.030
+Pi+V_5_	34.20^b^ ± 0.78	35.77^d^ ± 6.21	0.36^e^ ± 0.03	+V + Pi_5_	38.3^c^ ± 0.48	19.12^d^ ± 4.15	0.116^cde^ ± 0.032
+Pi+V_10_	26.10^c^ ± 0.99	42.07^c^ ± 8.56	2.01^c^ ± 0.30	+V + Pi_10_	40.2^b^ ± 0.63	15.09^de^ ± 11.65	0.076^cde^ ± 0.038
+Pi+V_15_	25.80^c^ ± 0.79	48.46^c^ ± 7.67	2.32^b^ ± 0.18	+V + Pi_15_	40.7^b^ ± 0.48	13.30^de^ ± 7.58	0.056^de^ ± 0.032
+V alone	43.80^d^ ± 0.63	6.42^e^ ± 11.47	0.07^g^ ± 0.18	+V alone	43.6^a^ ± 0.84	5.60^e^ ± 9.39	0.017^e^ ± 0.029
+Pi alone	25.20^f^ ± 0.79	62.57^a^ ± 3.01	2.72^a^ ± 0.16	+Pi alone	25.7^f^ ± 0.82	63.55^a^ ± 2.75	2.370^a^ ± 0.252
Absolute control	34.60^e^ ± 1.26	54.90^b^ ± 1.78	1.75^d^ ± 0.18	Absolute control	34.9^e^ ± 1.20	50.02^b^ ± 4.85	1.710^b^ ± 0.177
SE (m)	0.256	3.518	0.053	SE (m)	0.241	3.515	0.035
CD (0.05)	0.723	9.918	0.149	CD (0.05)	0.678	9.911	0.100

In pre-inoculation studies, *P. indica*-colonized bitter gourd seedlings (four leaf stage) were graft-transmitted with scions having ToLCV, PRSV, and CMV at 0 (+Pi/+V_0_), 2 (+Pi/+V_2_), 5 (+Pi/+V_5_), 10 (+Pi/+V_10_), and 15 (+Pi/+V_15_) days after the colonization. In post-inoculation studies, the virus-infected scions (ToLCV, PRSV, and CMV) were graft-transmitted to four leaf-staged healthy bitter gourd seedlings grown in pots filled with potting mixture. The virus-inoculated seedlings were then transplanted to pots filled with *P. indica*-multiplied potting mixture having 10^6^ cfu g^−1^ for fungal colonization at 0 (+V/+Pi_0_), 2 (+V/+Pi_2_), 5 (+V/+Pi_5_), 10 (+V/+Pi_10_) and 15 (+V/+Pi_15_) days after virus inoculation, as described in the Materials and Methods. The control seedlings (without *P. indica*) wedge-grafted with the virus-infected scion served as the positive control (+V alone), while *P. indica*-colonized seedlings wedge-grafted with virus-free scion served as negative control (+Pi alone). The seedlings wedge-grafted with the virus-free scion (without any treatment) served as the absolute control. ^*^Values are the mean of 50 replications ± standard deviation. Ten plants per treatment per experiment. Five independent experiments were conducted. Superscripts with same letters indicate on-par values, and those with different letters indicate a significant difference at the 5 percent level of significance. DAI: days after inoculation of the virus by grafting. Pooled CRD. SE: standard error. CD: critical difference at the 5% level of significance.

In the post-inoculation studies, the same trend of results observed in the pre-inoculation studies was noted. However, the performance of *P. indica* in managing the BGMC as a curative measure was not up to that of the prophylactic treatment. The bitter gourd plants inoculated with the viruses and colonized with *P. indica* on the same day, 2 DAI, and 5 DAI of the inoculation took less than 40 days to flower compared to 43.60 days in the infected control plants; whereas the *P. indica*-colonized plants started to flower at 25.70 days (Table 5). The virus inoculation drastically reduced the fruit set to 5.60 percent in the non-colonized plants. However, fruit setting was enhanced to 31.91, 19.16, and 19.12% when the plants were colonized with *P. indica* on the same day, 2 days, and 5 days after the virus inoculation, respectively. *Piriformospora indica*-colonized plants without the inoculation had the maximum fruit set (63.55%) and yield (2.37 kg plant^−1^) (Table 5). *Piriformospora indica* colonization in the inoculated plants improved the yield to 0.171 kg plant^−1^, 0.124 kg plant^−1^, and 0.116 kg plant^−1^ when colonized with *P. indica* on the same day, 2 days, and 5 days after the virus inoculation, respectively. The virus-inoculated plants without *P. indica* colonization recorded the lowest yield (0.017 kg plant^−1^). These results indicate the partial curative effect of the endophyte on the management of BGMC incited by ToLCV, PRSV, and CMV.

### 3.6 *Piriformospora indica* drastically reduced the incidence and severity of bitter gourd mosaic complex disease in the field

**Figure 7:**
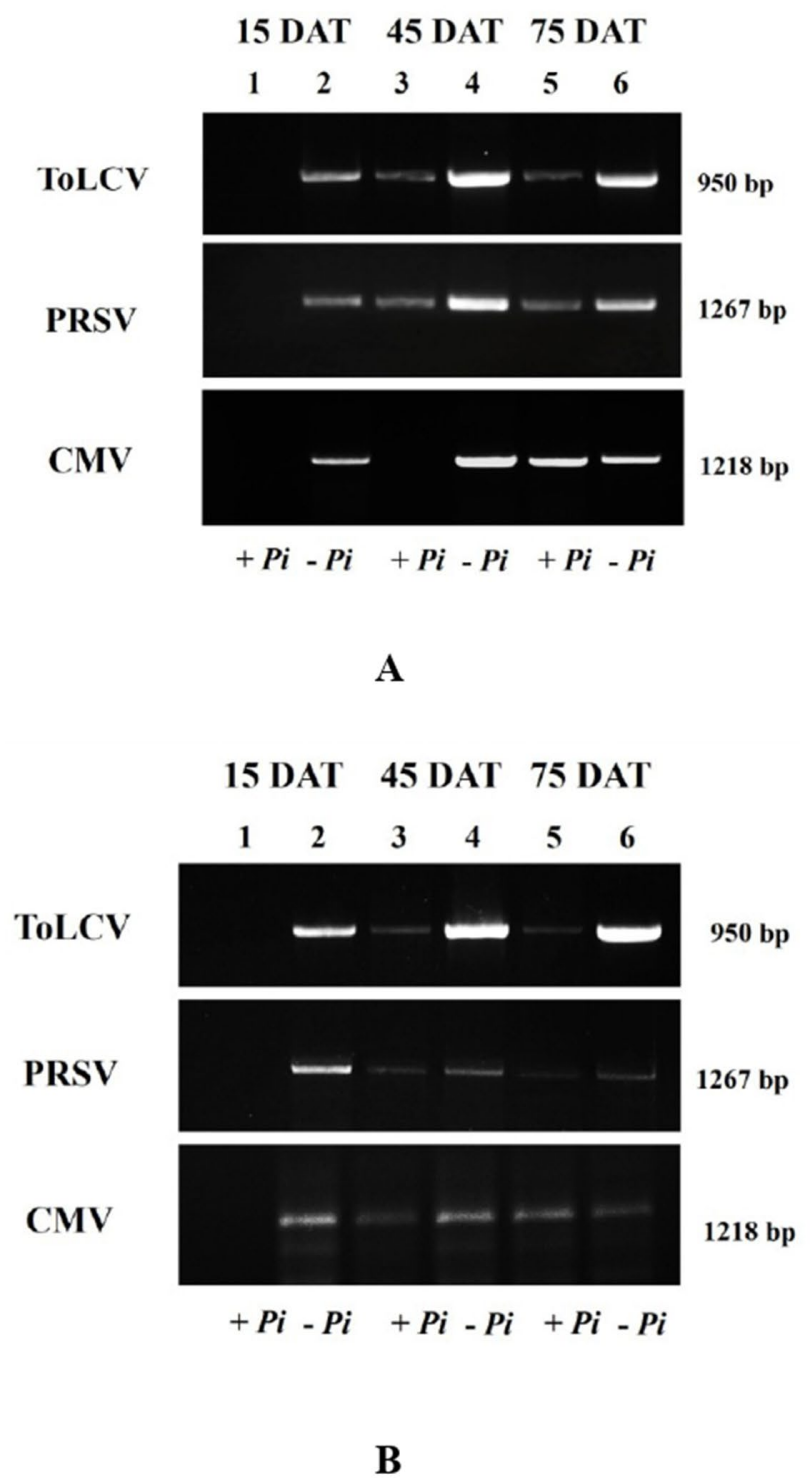
Electrophoresis gel image of amplified DNA of ToLCV and amplified cDNA of PRSV and CMV using respective coat protein specific primers in *P. indica*-colonized bitter gourd leaf samples at 15 DAT, 45 DAT, and 75 DAT from field during summer **(A)** and rabi **(B)** seasons. Fifteen-day-old *P. indica*-colonized and control bitter gourd seedlings, grown as per the procedure mentioned in materials and methods, were transplanted to the pits @ 3 seedlings per pit for both treatments. 1 kb DNA ladder; + Pi—*P. indica*-colonized plants and—Pi—control plants.

The results of the pot culture experiments were further validated in the field in both summer and rabi seasons for 3 years (Supplementary Figure 6). During the summer season, the symptoms of BGMC (natural incidence) first appeared in the control plants by 12.80 days (Table 6). *Piriformospora indica* colonization significantly delayed the symptom appearance to 28.27 days. Similar results were observed in the rabi field as well. Symptoms appeared within 17.6 days in control plants, whereas the colonized plants showed symptoms only after 30.53 days (Table 6). During summer, at 15 DAT, *P. indica*-colonized plants showed no symptoms of BGMC disease, whereas an incidence of 42.22% was recorded in the control plants (Table 7). At 45 DAT, DI of 51.11% was recorded in the colonized plants compared to 100 per cent incidence in control plants. In the rabi season, DI of 35.55% was recorded in the colonized plants over DI of 68.89% in control plants at 45 DAT. The incidence was found to be 77.78% in the summer season and 64.45% in the rabi season for *P. indica*-colonized plants at 75 DAT, whereas control plants in both seasons recorded 100 per cent disease incidence. Similarly, BGMC severity in terms of VI was recorded as 0, 12.96, and 25.18 in the *P. indica*-colonized plants, and 1.48, 27.78, and 75.53 in control plants at 15, 45, and 75 DAT, respectively, (Table 7). During the rabi season, VI recorded was 0, 12.59, and 28.90 in the *P. indica* colonized plants, and 2.96, 39.63, and 71.11 in control plants at 15 DAT, 45 DAT, and 75 DAT, respectively, (Table 7). The diseased control plants showed different symptoms of BGMC in severe form, whereas mild to moderate levels of symptoms were observed in the *P. indica*-colonized plants in summer and rabi. However, *P. indica* colonization drastically reduced the incidence and severity of BGMC in both seasons. Further, serological and molecular methods confirmed a low titer value of ToLCV, PRSV, and CMV in the *P. indica*-colonized plants compared to the control plants (Figure 7; Supplementary Table S7 and S8).

**Table 6:** Effect of *P. indica* colonization in bitter gourd plants on days taken for symptom development by natural incidence of bitter gourd mosaic complex disease incited by the combined infection of ToLCV, PRSV, and CMV under field conditions during summer and rabi seasons.

Treatments	Days taken for BGMC symptom development	
	Summer^*^	Rabi^*^
+ *P. indica*	28.27^a^ ± 4.80	30.53^a^ ± 2.01
Control	12.80^b^ ± 3.60	17.60^b^ ± 3.56
*t*-value	19.59	15.64
*t*-value (0.05)	2.001	

Fifteen-day-old P. indica-colonized and control bitter gourd seedlings, grown as per the procedure mentioned in materials and methods, were transplanted to the pits at three seedlings per pit for both treatments. ^*^Values are the mean of 60 replications ± standard deviation. Three independent experiments, each under summer and rabi seasons, were conducted. Twenty replication per treatment per experiment. Superscripts with same letters indicate on-par values, and those with different letters indicate a significant difference at the 5 percent level of significance.

**Table 7:** Effect of *P. indica* colonization in bitter gourd plants on natural incidence and severity of bitter gourd mosaic complex disease under field condition incited by the combined infection of ToLCV, PRSV, and CMV during summer and rabi seasons.

DAT	Disease incidence (%)^*^						Disease severity as vulnerability index^*^					
	Summer			Rabi			Summer			Rabi		
	+ *P. indica*	Control	*t*-value	+ *P. indica*	Control	*t*-value	+ *P. indica*	Control	*t*-value	+ *P. indica*	Control	*t*-value
15	0^a^	42.22^b^ ± 3.85	18.99	0^a^	40.00 ± 6.66	10.39	0^a^	1.48 ± 0.30	8.53	0^a^	2.96 ± 0.21	14.26
45	51.11^a^ ± 3.84	100^b^ ± 0.00	22.02	35.55^a^ ± 3.25	68.89 ± 3.84	18.66	12.96^a^ ± 0.64	27.78 **±** 1.11	20.03	12.59^a^ ± 0.64	39.63 ± 0.64	17.54
75	77.78^a^ ± 3.05	100^b^ ± 0.00	10.00	64.45^a^ ± 3.42	100 ± 0.00	15.99	35.18^a^ ± 3.16	75.53 ± 1.97	18.67	28.90^a^ ± 0.71	71.11 ± 1.68	24.34
*t*-value (0.05)	2.001											

Fifteen-day-old *P. indica*-colonized and control bitter gourd seedlings, grown as per the procedure mentioned in materials and methods, were transplanted to the pits at three seedlings per pit for both treatments. ^*^Values are the mean of 60 replications ± standard deviation. Three independent experiments, each under summer and rabi seasons, were conducted. Twenty replication per treatment per experiment. DAT, days after transplanting.

Biometric parameters such as the number of days taken for flowering, percent fruit set, and yield were also compared through the field studies (Table 8). As in pot culture studies, *P. indica* promoted early flowering in bitter gourd plants. The *P. indica*-colonized plants took only 24.47–28.06 days to start flowering compared to 33.86–35.86 days in control plants. *Piriformospora indica* colonization also enhanced fruit set to 71.36–74.95% compared to 42.60–43.27% in control plants (Table 8). A significant difference in yield was also observed in the colonized and non-colonized plants irrespective of the multiple virus inoculation. The yield recorded was 2.89 kg plant^−1^ in the *P. indica*-colonized plants and 0.81 kg plant^−1^ in control plants in summer, and 3.07 kg plant^−1^ in the colonized plants against 0.82 kg plant^−1^ in control plants during rabi (Table 8; Supplementary Figure 7).

**Table 8:** Effect of *P. indica* colonization in bitter gourd plants on yield parameters under field condition on natural incidences of bitter gourd mosaic complex disease incited by the combined infection of ToLCV, PRSV, and CMV during summer and rabi seasons.

Sl. No.	Parameters	Summer^*^			Rabi^*^		
		+ *P. indica*	Control	*t*-value	+ *P. indica*	Control	*t*-value
1	Days taken for flowering (DAT)	24.47^a^ ± 3.65	33.86^b^ ± 4.76	16.00	28.06^a^ ± 2.30	35.86^b^ ± 3.40	12.04
2	Fruit setting (%)	71.36^a^ ± 12.06	43.27^b^ ± 9.20	13.18	74.95^a^ ± 7.43	42.60^b^ ± 10.09	15.75
3	Average yield (kg plant^−1^)	2.89^a^ ± 0.23	0.81^b^ ± 0.34	16.22	3.07^a^ ± 0.25	0.82^b^ ± 0.16	29.90
*t*-value (0.05)		2.001					

Fifteen-day-old *P. indica*-colonized and control bitter gourd seedlings, grown as per the procedure mentioned in materials and methods, were transplanted to the pits at three seedlings per pit for both treatments. ^*^Values are the mean of 60 replications ± standard deviation. Three independent experiments, each under summer and rabi seasons, were conducted. Twenty replication per treatment per experiment. Superscripts with same letters indicate on-par values, and those with different letters indicate a significant difference at the 5 percent level of significance.

The findings from the field experiments clearly showed that the *P. indica*-colonized plants displayed lower disease incidence and severity and exhibited better growth and yield parameters compared to the control plants. Both pot culture and field studies revealed that *P. indica* could successfully manage the simultaneous infection of both DNA and RNA viruses, which belong to three different families: *Geminiviridae* (ToLCV), *Potyviridae* (PRSV), and *Bromoviridae* (CMV). Remarkably, *P. indica* reduced the severity of these infections without compromising growth and yield, showcasing its broad-spectrum efficacy in managing complex viral infections.

### 3.7 *Piriformospora indica* colonization significantly reduced the reactive oxygen species accumulation in BGMC infected bitter gourd

**Figure 8:**
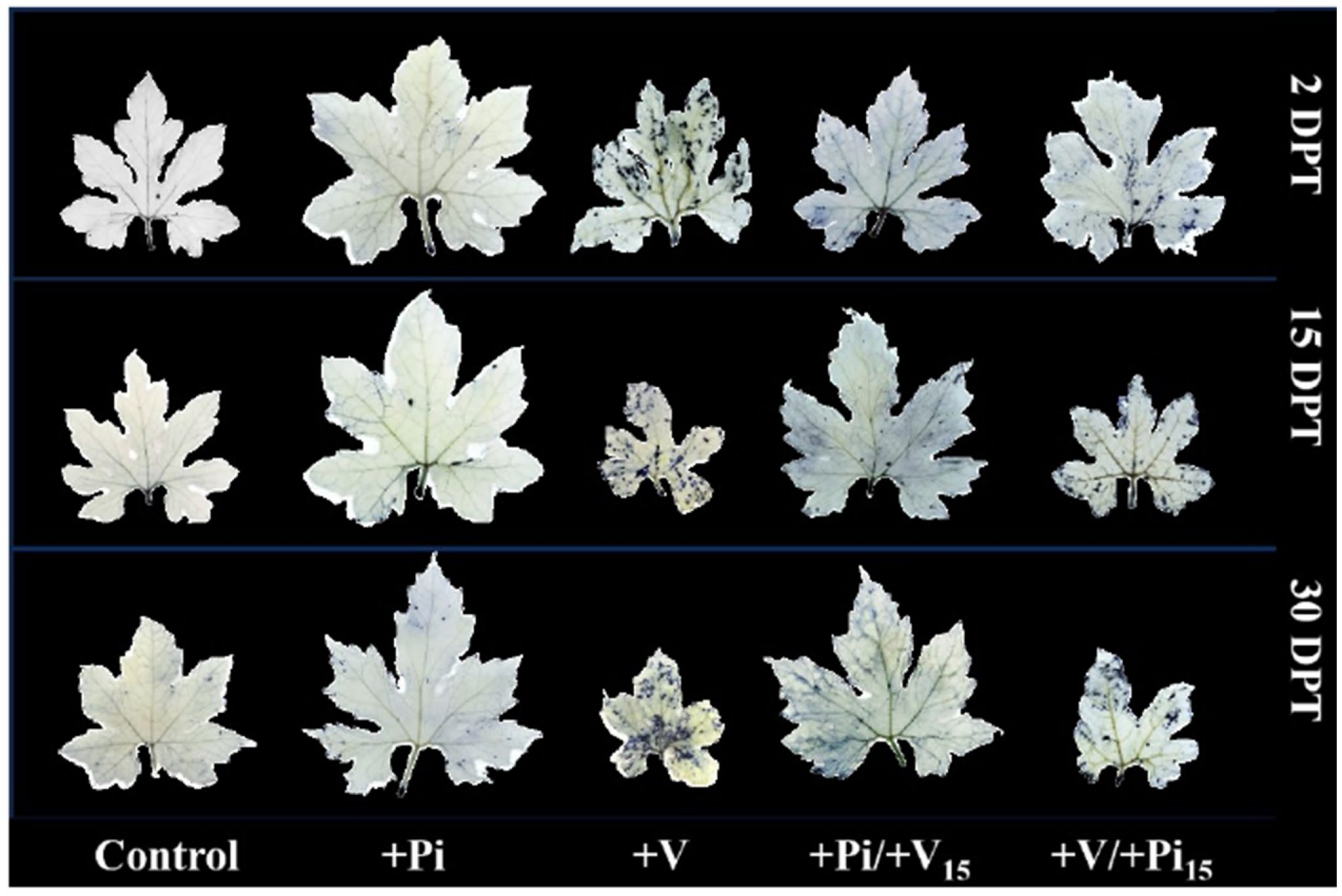
Effect of *P. indica*-colonization on reactive oxygen species (ROS) accumulation in bitter gourd leaves on pre- and post- inoculation of ToLCV, PRSV, and CMV causing bitter gourd mosaic complex at 2 DPT, 15 DPT, and 30 DPT after NBT staining. *Piriformospora indica*-colonized bitter gourd seedlings (4 leaf-stage) were graft-transmitted with scions having the viruses at 15 days after the colonization (+Pi/+V_15_) as described in the materials and methods. The viruses-infected scions were graft-transmitted to 4 leaf-stage healthy bitter gourd seedlings grown in pots filled with potting mixture; and then the viruses-inoculated-seedlings were transplanted to pots filled with *P. indica*- multiplied potting mixture having 10^6^ cfu g^-1^ for the fungal colonization at 15 days after the viruses inoculation (+V + Pi_15_) as described in the materials and methods. The control seedlings (without *P. indica*) wedge grafted with the viruses-infected scion was positive control (+V) and *P. indica*- colonized seedlings wedge grafted with viruses-free scion served as negative control (+Pi). The seedlings wedge grafted with viruses-free scion (without any treatment) served as control. Ten plants per treatment. Representative picture of five independent experiments are shown. DAI, days after inoculation of the viruses by grafting; DPT, Days after post treatments.

**Figure 9:**
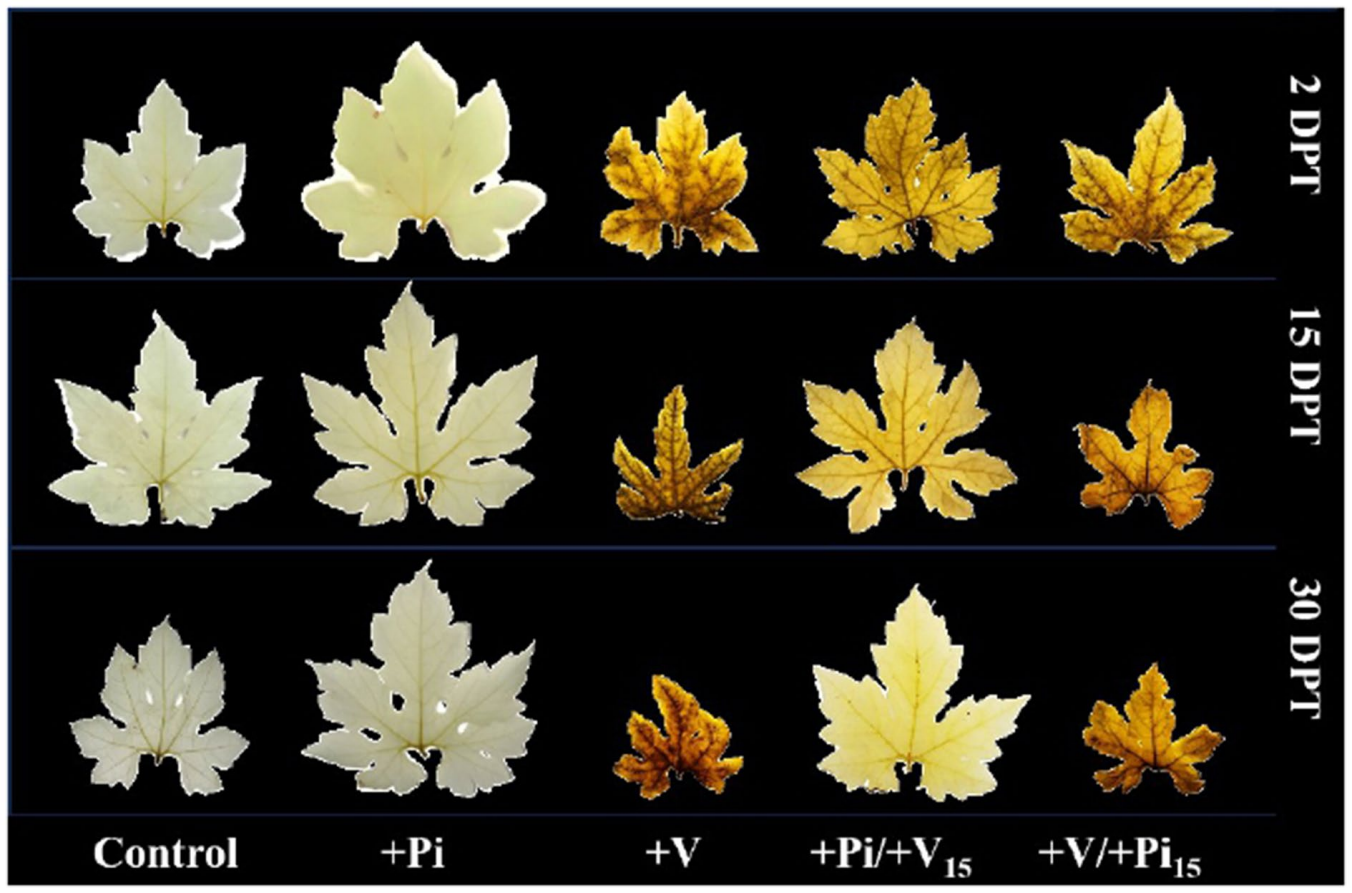
Effect of *P. indica*-root colonization on hydrogen peroxide (H_2_O_2_) accumulation in bitter gourd leaves on pre- and post- inoculation of ToLCV, PRSV, and CMV causing bitter gourd mosaic complex at 2 DPT, 15 DPT, and 30 DPT after DAB staining. *P. indica*-colonized bitter gourd seedlings (4 leaf-stage) were graft-transmitted with scions having the viruses at 15 days after the colonization (+Pi/+V_15_) as described in the materials and methods. The viruses-infected scions were graft-transmitted to 4 leaf-stage healthy bitter gourd seedlings grown in pots filled with potting mixture; and then the viruses-inoculated-seedlings were transplanted to pots filled with *P. indica*- multiplied potting mixture having 10^6^ cfu g^-1^ for the fungal colonization at 15 days after the viruses inoculation (+V +Pi_15_) as described in the materials and methods. The control seedlings (without *P. indica*) wedge grafted with the viruses-infected scion was positive control (+V) and *P. indica*- colonized seedlings wedge grafted with viruses-free scion served as negative control (+Pi). The seedlings wedge grafted with viruses-free scion (without any treatment) served as control. Ten plants per treatment. Representative picture of five independent experiments are shown. DAI, Days after inoculation of the viruses by grafting; DPT, Days after post treatments.

We further examined how the reactive oxygen species and antioxidant enzymes were altered in the *P. indica* - bitter gourd - multiple virus interaction at 2, 15, and 30 days after pre- and post-inoculation studies. The accumulation of ROS and H_2_O_2_ was higher in ToLCV, PRSV, and CMV inoculated plants compared to *P. indica*-colonized and absolute control plants at all intervals (Figures 8 and 9). The accumulation of ROS and H_2_O_2_ in leaves gradually increased in the subsequent stages of plant growth, as evidenced by the increase in color intensity. The plants inoculated with the viruses after 15 days of *P. indica* colonization showed a significant level of ROS and H_2_O_2_ accumulation in leaves at 2 days after post-treatment (DPT), but the intensity gradually reduced in later stages. The plants inoculated with the viruses and then colonized with *P. indica* after 15 days of the multiple virus inoculation showed a higher accumulation of ROS and H_2_O_2_, but the level of accumulation was low compared to the viruses alone inoculated plants (Figures 8 and 9). *Piriformospora indica* colonization at different stages of the crop was also confirmed using Pi-tef primers at the molecular level in both pre- and post-inoculation studies (Supplementary Figure 8). These results indicated that *P. indica* colonization significantly reduced the ROS and H_2_O_2_ accumulation in the multiple virus infected bitter gourd plants.

### 3.8 *Piriformospora indica* enhanced activities of antioxidant enzymes in BGMC infected bitter gourd plants

**Figure 10:**
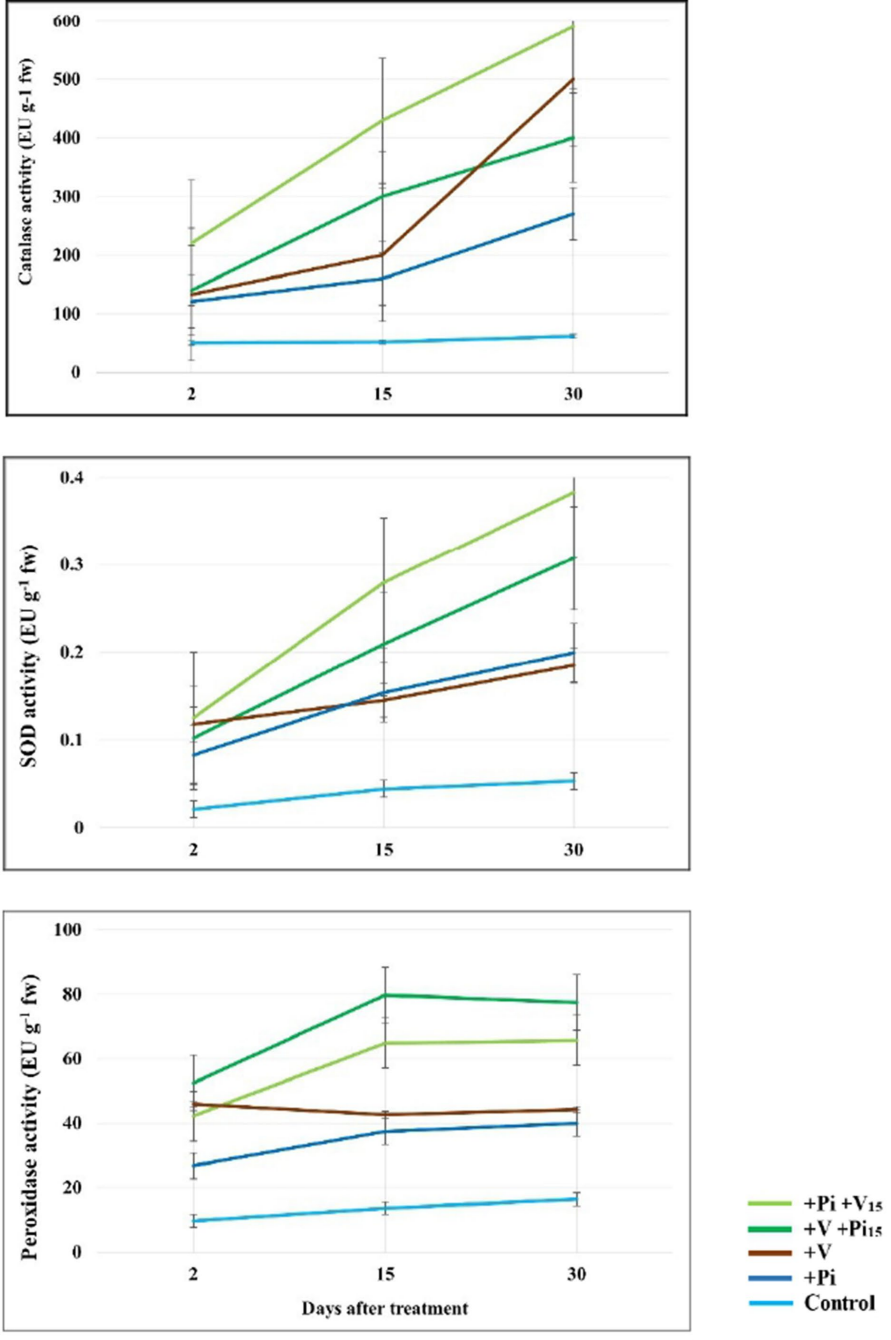
Changes in catalase, superoxide dismutase (SOD), and peroxidase (PO) activity in bitter gourd leaves of *P. indica*-colonized plants on pre- and post- inoculation of ToLCV, PRSV, and CMV causing bitter gourd mosaic complex at 2 DPT, 15 DPT, and 30 DPT. *Piriformospora indica*-colonized bitter gourd seedlings (4 leaf-stage) were graft-transmitted with scions having the viruses at 15 days after the colonization (+Pi/+V_15_). The viruses-infected scions were graft-transmitted to 4 leaf-stage healthy bitter gourd seedlings grown in pots filled with potting mixture; and then the viruses-inoculated- seedlings were transplanted to pots filled with *P. indica*-multiplied potting mixture having 10^6^ cfu g^−1^ for the fungal colonization at 15 days after the viruses inoculation (+V +Pi_15_). The control seedlings (without *P. indica*) wedge grafted with the viruses-infected scion was positive control (+V) and *P. indica*-colonized seedlings wedge grafted with viruses-free scion served as negative control (+Pi). The seedlings wedge grafted with viruses-free scion (without any treatment) plants per treatment. Five independent experiments were done.

The catalase, SOD, and PO activities were found to be increasing in all treatments over time. Among the different intervals such as 2 DPT, 15 DPT, and 30 DPT, the highest activity of catalase was found in the *P. indica*-colonized plants inoculated with ToLCV, PRSV, and CMV at 15 DAC (up to 590.20 EU g^−1^ fw) and was followed by the plants inoculated with the viruses and colonized with *P. indica* at 15 DAI (up to 500.00 EU g^−1^ fw) (Figure 10A). The virus-inoculated plants and *P. indica*-colonized plants had comparatively lower enzyme activity. The lowest activity was observed in the absolute control at all intervals. A similar trend was also recorded with SOD activity. At 2 DPT, the highest activity of SOD was observed in *P. indica*-colonized plants graft-inoculated with the viruses after 15 DAC (0.125 EU g^−1^ fw), followed by the virus-inoculated control (0.118 EU g^−1^ fw) and the virus-inoculated plants colonized by *P. indica* at 15 DAI (0.102 EU g^−1^ fw) (Figure 10B). A similar trend was observed at 15 DPT and 30 DPT. *Piriformospora indica*-colonized plants and absolute control plants had the lowest SOD activity.

Peroxidase activity also showed drastic changes when *P. indica*-colonized plants were pre- and post-inoculated with multiple viruses compared to the negative and positive control plants. In general, the highest activity of peroxidase was observed in plants graft-inoculated with the viruses and colonized with *P. indica* at 15 DAI (Figure 10C). At 2 DPT, 15 DPT, and 30 DPT, significantly higher peroxidase activity was found in the inoculated plants colonized by *P. indica* at 15 DAI compared to the virus-inoculated plants (up to 79.70 EU g^−1^ fw). A significant increase in peroxidase enzyme activity was observed in the plants colonized with *P. indica* and graft-inoculated with the viruses after 15 DAC. This trend was recorded in plants at 30 DPT, and the highest enzyme activity was observed in plants inoculated with viruses and colonized with *P. indica* after 15 DAI (77.43 EU g^−1^ fw), followed by *P. indica*-colonized plants graft-inoculated with the viruses (65.68 EU g^−1^ fw). The least PO activity was recorded in the plants grafted with the infected scion (44.10 EU g^−1^ fw) and *P. indica*-colonized plants (40.00 EU g^−1^ fw) (Figure 10C). Hence, *P. indica* colonization significantly enhanced the activities of key antioxidant enzymes such as catalase, SOD, and PO in bitter gourd plants infected with ToLCV, PRSV, and CMV.

### 3.9 *Piriformospora indica*-mediated remission of BGMC symptoms was effected through downregulation of ROS markers and upregulation of antioxidant genes in the nucleus, mitochondria, and chloroplast

**Figure 11:**
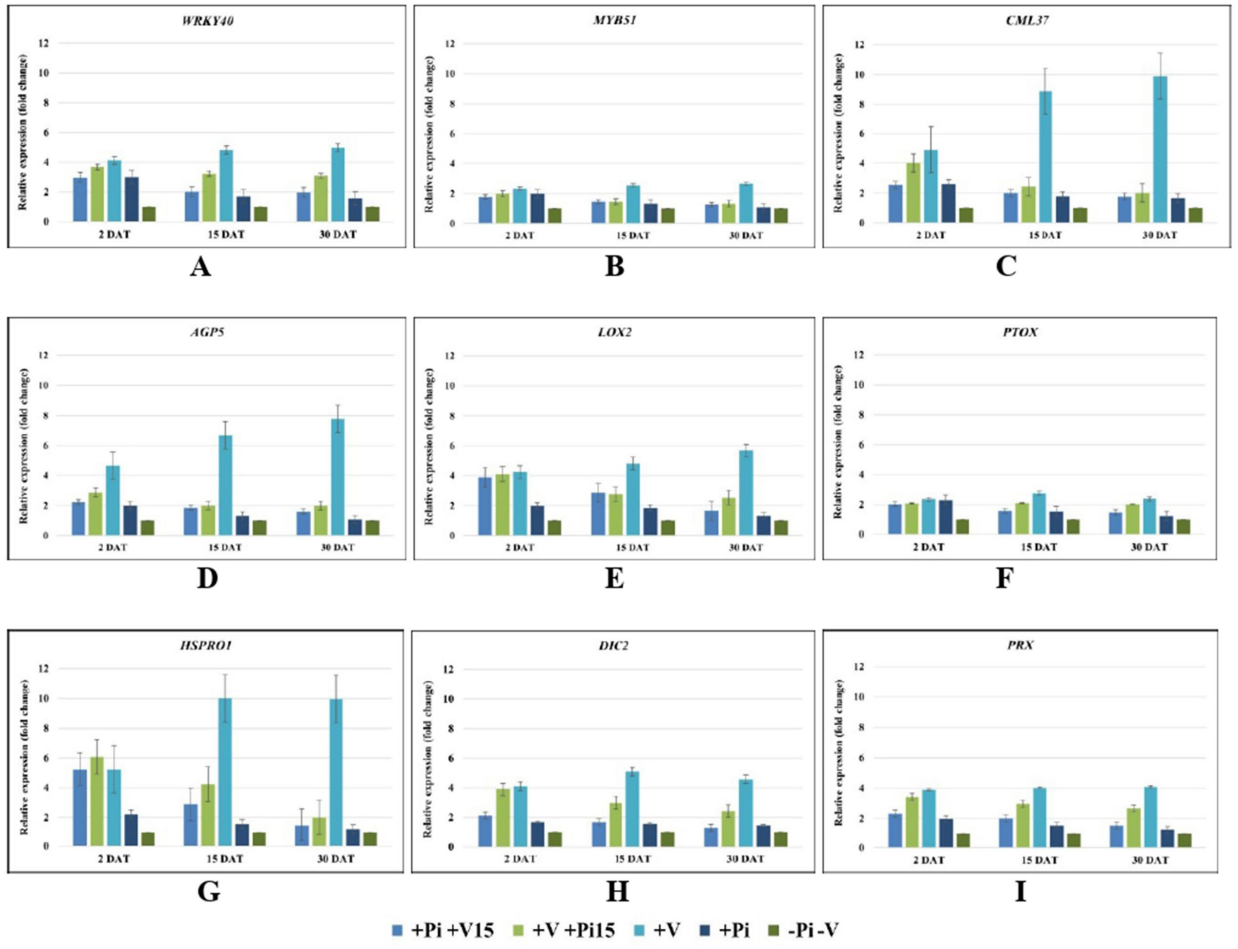
Relative gene expression by quantitative real-time PCR (qRT-PCR) of ROS and H_2_O_2_ marker genes localized in nucleus (ROS–WRKY40 **(A)**, MYB51 **(B)**, CML37 **(C)**; H_2_O_2_–AGP5 **(D)**), chloroplast (ROS–LOX2 **(E)**; H_2_O_2_–PTOX **(F)**), and mitochondria (ROS–HSPRO1 **(G)**, DIC2 **(H)**; H_2_O_2_–PRX **(I)**) of bitter gourd leaves of *P. indica*-colonized plants on pre- and post-inoculation of ToLCV, PRSV, and CMV causing bitter gourd mosaic complex at 2 DPT, 15 DPT, and 30 DPT. *Piriformospora indica*-colonized bitter gourd seedlings (4 leaf-stage) were graft-transmitted with scions having the viruses at 15 days after the colonization (+Pi/+V_15_). The viruses-infected scions were graft-transmitted to 4 leaf-stage healthy bitter gourd seedlings grown in pots filled with potting mixture; and then the viruses-inoculated-seedlings were transplanted to pots filled with *P. indica*- multiplied potting mixture having 10^6^ cfu g^-1^ for the fungal colonization at 15 days after the viruses inoculation (+V+Pi_15_). The control seedlings (without *P. indica*) wedge grafted with the viruses-infected scion was positive control (+V) and *P. indica*-colonized seedlings wedge grafted with viruses-free scion served as negative control (+Pi). The seedlings wedge grafted with viruses-free scion (without any treatment) served as control (−PI−V). Ten plants per treatment. Five independent experiments were done. Relative transcript levels were calculated using the 2-44Ct method, normalized against the house keeping gene GAPDH. Error bars represent ± standard error of the mean from three biological replicates. Significant differences ANOVA followed by LSD test (*p* ≤ 0.05).

The transcript levels of ROS and H_2_O_2_ marker and antioxidant genes involved in *P. indica*-mediated tolerance to bitter gourd mosaic complex were assessed through quantitative RT-PCR (qRT-PCR) using gene-specific primers. The relative expressions of selected marker genes localized in the nucleus, chloroplast, and mitochondria were studied. qRT-PCR studies on the relative expressions of different ROS and H_2_O_2_ marker genes showed that the ROS marker genes located in the nucleus (*WRKY40*, *MYB51*, *CML37*, *AGP5*), chloroplast (*LOX2*, *PTOX*), and mitochondria (*HSPRO1*, *DIC2*, *PRX*) were significantly downregulated compared to the virus-inoculated control (Figure 11). In contrast, the virus-inoculated control plants showed up to 10-fold upregulation of these oxidative stress marker genes, reflecting higher ROS accumulation in cells under viral pressure. The regulation of different ROS and H_2_O_2_ marker genes present in the nucleus, chloroplast, and mitochondria confirms that the interaction of *P. indica* with the plant generates retrograde signaling molecule(s) that can simultaneously modulate the genes present in these organelles.

**Figure 12:**
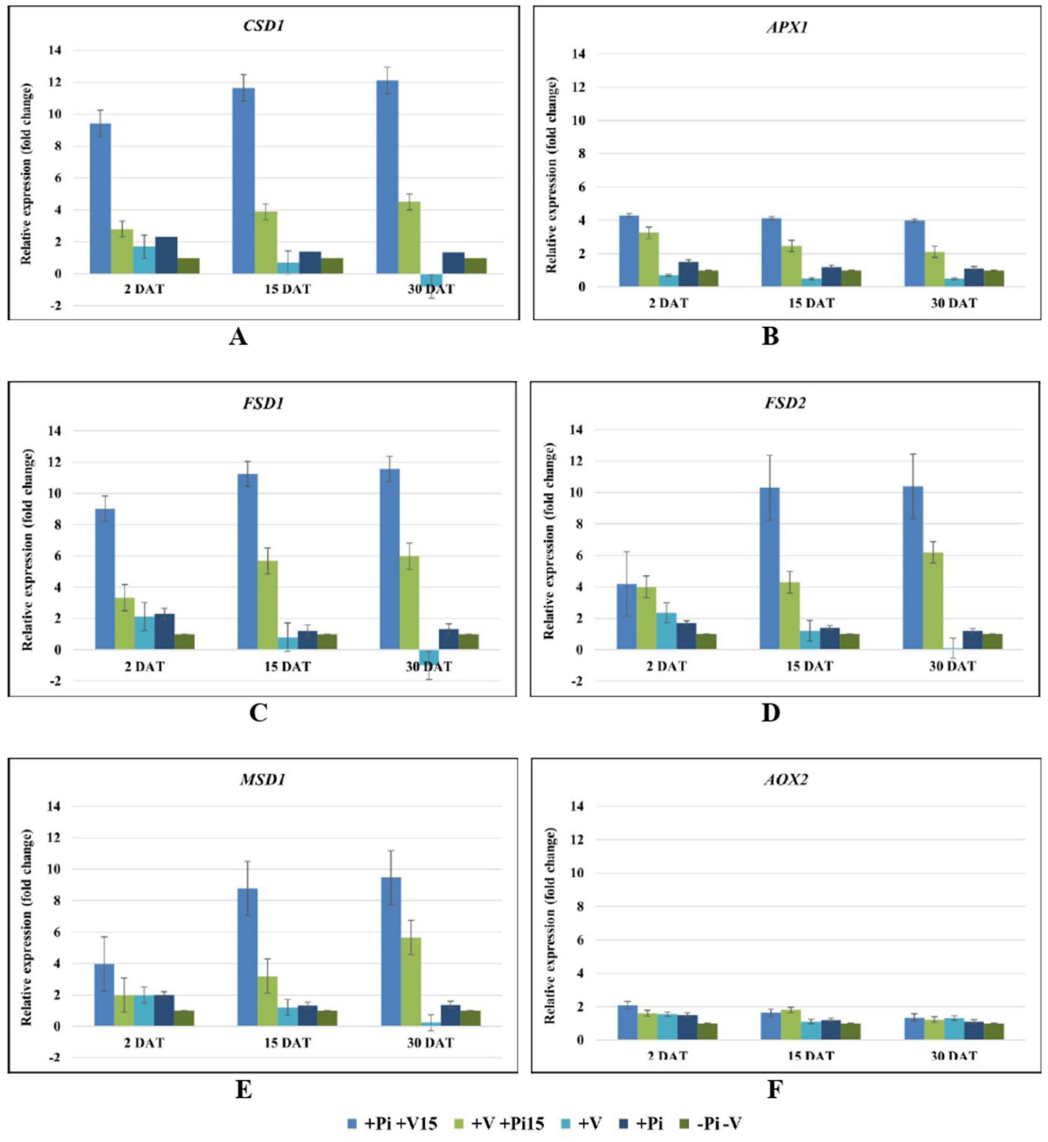
Relative gene expression by quantitative real-time PCR (qRT-PCR) of antioxidant enzyme genes localized in nucleus (A: *CSD1*, B: *APX1*), chloroplast (C: *FSD1*, D: *FSD2*), and mitochondria (E: *MSD1*, F: *AOX2*) of bitter gourd leaves of *P. indica*-colonized plants on pre- and post-inoculation of ToLCV, PRSV and CMV causing bitter gourd mosaic complex at 2 DPT, 15 DPT, and 30 DPT. *Piriformospora indica*-colonized bitter gourd seedlings (4 leaf-stage) were graft-transmitted with scions having the viruses at 15 days after the colonization (+Pi/+V_15_). The viruses-infected scions were graft-transmitted to 4 leaf-stage healthy bitter gourd seedlings grown in pots filled with potting mixture; and then the viruses-inoculated-seedlings were transplanted to pots filled with *P. indica.* multiplied potting mixture having 106 cfu g^-1^ for the fungal colonization at 15 days after the viruses inoculation (+V+Pi_15_). The control seedlings (without *P. indica*) wedge-grafted with the viruses-infected scion was positive control (+V) and *P. indica*-colonized seedlings wedge grafted with viruses-free scion served as negative control (+Pi). The seedlings wedge grafted with viruses-free scion (without any treatment) served as control (-PI-V). Ten plants per treatment. Five independent experiments were done. Relative transcript levels were calculated using the 2^-^ΔΔCt method, normalized against the house keeping gene GAPDH. Error bars represent ± standard error of the mean from three biological replicates. Significant differences between treatments were determined by ANOVA followed by LSD test (*p* ≤ 0.05).

On the contrary, ROS-scavenging genes encoding key antioxidant enzymes localized in the nucleus (*CSD1*, *APX1*), chloroplast (*FSD1*, *FSD2*), and mitochondria (*MSD1*, *AOX2*) were significantly upregulated in *P. indica*-colonized plants inoculated with ToLCV, PRSV, and CMV at 15 DAC (pre-inoculation), and the plants inoculated with the viruses and then colonized with *P. indica* after 15 days of the multiple virus inoculation (post-inoculation) (Figure 12). This enhancement was substantially lower in both the virus-inoculated control and non-inoculated controls. While *P. indica* colonized plants showed mild induction of antioxidant genes, the absolute control maintained baseline expression for all marker genes. Together, these results confirmed that *P. indica* not only suppressed ROS and H_2_O_2_ marker gene expressions upon viral challenge but also boosted the plant’s antioxidant machinery, thereby conferring enhanced oxidative stress tolerance in the infected bitter gourd plants.

## 4 Discussion

Viruses are biotrophic pathogens that rely on living host cells to survive and replicate. One of the most visible effects of viral infection is the degradation of chlorophyll, due to excess production and accumulation of different ROS, leading to characteristic symptoms such as discoloration and color change (Krishnan, 2021; Sinijadas, 2024; Chandran, 2025). These symptoms indicate impaired photosynthesis, which reduces the plant’s ability to produce energy for growth and reproduction. As a result, infected plants often show stunted growth, delayed flowering, and reduced fruit or seed yield. Plant viruses act as metabolic parasites, exploiting host resources to ensure their own replication while affecting the plant’s growth and development. This biotrophic strategy allows viruses to persist in living tissues without killing the host, ensuring their spread to new plants through vectors like insects, mechanical damage, or seeds.

*Piriformospora indica* is a beneficial fungal root endophyte that forms a symbiotic association with a wide range of plants (Varma et al., 2012; Franken, 2012; Gill et al., 2016). It colonizes the roots without causing any visible symptoms and enhances plant growth, nutrient uptake, and the overall performance of crop plants (c.f. Oelmüller et al., 2009; Johnson et al., 2014b; Ibrahim et al., 2025). This mutualistic interaction helps plants to tolerate biotic and abiotic stresses. One of the key mechanisms behind this stress tolerance is the regulation of reactive oxygen species. Under stress conditions, plants often produce excessive ROS, which can damage cell membranes, proteins, and DNA (Torres, 2010; Mittler et al., 2011). *Piriformospora indica* reduces this damage by modulating ROS generation and activating the plant’s antioxidant defense system (Matsuo et al., 2015). Studies have shown that plants colonized by *P. indica* exhibit higher activity of antioxidant enzymes such as superoxide dismutase, catalase, and peroxidases (Sun et al., 2010). These enzymes neutralize harmful ROS, preventing oxidative stress and maintaining cellular health (Mittler et al., 2004). In addition to oxidative stress management, *P. indica* improves nutrient acquisition by enhancing root surface area (Lee et al., 2011) and promoting the uptake of key minerals like phosphorus (Ngwene et al., 2016), nitrogen (Sherameti et al., 2005), and micronutrients (Sinijadas et al., 2023). The fungus also influences plant hormonal balance, increasing levels of growth-promoting hormones like auxins, cytokinins, and gibberellins (Vadassery et al., 2008; Xu et al., 2018), while fine-tuning stress-related hormones like abscisic acid (Peskan-Berghöfer et al., 2015). This dual role—promoting growth and reducing stress—makes *P. indica* a highly effective biological agent for sustainable agriculture. Through these mechanisms, *P. indica* acts as a plant growth-promoting fungus and a biological stress mitigator, helping plants remain healthy, productive, and resilient even under challenging environmental and pathogenic pressures.

Bitter gourd plants are well known for producing antimicrobial enzymes and bioactive compounds in almost all parts of the plant, including leaves, stems, fruits, and roots. These natural defense compounds, such as phenolics, flavonoids, alkaloids, and various pathogenesis-related proteins, normally act as a barrier against microbial colonization. The establishment of *P. indica* in bitter gourd roots was confirmed through staining, with early hyphal penetration detected from the 3^rd^ day after germination. The symbiosis promoted early seedling vigor and improved growth. Despite the strong antimicrobial interaction, *P. indica* is able to successfully colonize the roots of bitter gourd plants. This ability to thrive in a chemically defensive plant environment suggests that *P. indica* is highly adaptable and capable of colonizing even medicinally important crops like bitter gourd, which are naturally rich in antimicrobial metabolites.

Bitter gourd is one of the most popular and extensively cultivated vegetable crops in Kerala, India. Despite its adaptability and nutritional value, bitter gourd cultivation faces significant challenges due to various biotic and abiotic stresses. The incidence of an array of viral diseases is the major production constraint faced by farmers, especially after the great floods of 2018 and 2019 in Kerala. The congenial climatic conditions for the multiplication of insect vectors of the viruses and the susceptibility of cultivated varieties to multiple viruses contribute to the higher rate of incidence of viral diseases in Kerala. Bitter gourd mosaic complex is a group of viral diseases caused by multiple viruses in bitter gourd plants. It is an emerging field problem leading to complete crop loss. Many viruses have been reported to cause BGMC globally and nationally, of which tomato leaf curl virus (Begomovirus), papaya ringspot virus (Potyvirus), and cucumber mosaic virus (Bromovirus) were mainly reported to cause BGMC in Kerala (Radhika and Umamaheswaran, 2017).

This work marks the first in-depth study of utilizing the beneficial fungal root endophyte, *P. indica*, as a strategy for managing multiple or complex viral infections, offering an innovative solution for sustainable disease management in crops. The prophylactic use of *P. indica* as a biological control agent against BGMC was evaluated under pot culture conditions. *Piriformospora indica* colonization inhibited BGMC and delayed the expression of symptoms. The colonized plants showed reduced disease incidence as well as severity and enhanced growth and yield parameters in the infected plants. Studies conducted by Wang et al. (2015), Chandran et al. (2021), Sam (2021), Krishnan et al. (2022a), and Sinijadas et al. (2024) in other crops against single virus infections support our findings. The appearance of the symptoms was significantly delayed in the plants colonized with *P. indica* and graft-inoculated with the viruses at 15 DAC. This delay suggests that *P. indica* plays a crucial role in inhibiting early virus infection and symptom development. Earlier reports by Wang et al. (2015), Nair et al. (2024), and Sinijadas et al. (2024) also revealed the effect of *P. indica* colonization in inhibiting and delaying symptom expression in tomato plants infected with ToLCV, chili plants infected with chili leaf curl virus (ChiLCV), and banana plants infected with banana bract mosaic virus (BBrMV), respectively. Moreover, the severity of symptoms in the *P. indica*-colonized plants was significantly lower than in control plants. The incidence of viral disease was significantly lower in the *P. indica*-colonized plants compared to non-colonized control plants, with notable remission of symptoms. The remission of symptoms suggests that *P. indica* inhibits the virus replication or movement, or both, and enhances the plant defense mechanisms, possibly by priming the host immune responses.

A key finding of this study was the reduction in viral titers in the *P. indica*-colonized plants, which was evident in both serological and molecular assays. This reduction in viral titer was consistent with the gradual remission of symptoms observed in the treatments. The ability of *P. indica* to reduce viral titers is likely due to its ability to inhibit the replication, movement, and infectivity of the viruses; enhance host defense systems mediated by signaling cascades involving secondary messengers such as cytosolic calcium, reactive oxygen species, and other defense-related signaling molecules; and upregulate antioxidant enzymes (Johnson et al., 2014b, 2018). Sam (2021), Safeer and Thara (2022), Krishnan (2021), Sinijadas et al. (2023, 2024), and Nair et al. (2024) have earlier reported the potential of *P. indica* in managing single virus infections in tomato, orchids, pepper, banana, and chili, respectively. The direct antiviral activity of *P. indica* was demonstrated using water-diffusible exudates of *P. indica* (Pi-WDE). Before and after the sap transmission of blackeye cowpea mosaic virus (BlCMV—*Potyviridae*) on *Chenopodium amaranticolor* leaves, Pi-WDE, obtained on the 3rd day of growth of the *P. indica* mycelial mat, significantly reduced the local lesions to 4.33 compared to 18.0 lesions per leaf in the positive control (virus-alone treated leaves) (Chandran, 2019). It was also reported that the severity of blackeye cowpea mosaic disease was reduced to nearly 50% in Pi-WDE-treated plants on pre- and post-inoculation of the virus in vegetable cowpea or yard-long bean plants (*Vigna unguiculata* var. *sesquipedalis*). Molecular sieve studies indicated that small peptide(s) of less than 10 KDa present in Pi-WDE were responsible for the inhibition of the virus (Chandran, 2019).

From the observed symptoms, it can be inferred that the primary target of all three viruses is chlorophyll biosynthesis and degradation. The characteristic symptoms induced by these viruses appear to result from chlorophyll degradation. Interestingly, *P. indica* colonization significantly reduced symptom expression in infected plants. This reduction may be due to either the suppression of chlorophyll degradation or the enhancement of chlorophyll biosynthesis. Experimental findings confirm that in *P. indica*-colonized plants infected with the viruses, chlorophyll degradation was minimized while chlorophyll biosynthesis was simultaneously promoted (Krishnan, 2021; Sinijadas et al., 2024). This suggests that *P. indica* employs a dual strategy: reducing chlorophyll breakdown and increasing chlorophyll synthesis to maintain photosynthetic capacity and cellular functions, thereby supporting its mutualistic association with the host. Interestingly, the viruses may also benefit from this approach, as maintaining living host tissue is advantageous for their survival as biotrophic pathogens. This could explain why viral titer remains detectable even in the mild to absence of symptom expression.

In addition to its role in disease management, *P. indica* colonization significantly improved the growth and yield of bitter gourd plants, even under multiple virus infections. The *P. indica*-colonized plants enhanced vegetative growth, flowering, fruit set, and yield compared to the non-colonized virus-infected controls. Surprisingly, *P. indica*-colonized plants inoculated with the viruses at 15 DAC recorded the highest yield compared to the virus-inoculated plants. The increase in yield and other biometric parameters indicates that *P. indica* not only mitigates symptoms and crop loss due to viral disease but also promotes plant vigor. Studies by Oelmüller et al. (2009) and Johnson (2014) suggest that *P. indica* promotes the efficient absorption of essential nutrients, namely phosphorus and nitrogen, while also influencing plant hormones, leading to improved growth, stress resilience, and overall performance of the plant. Hence, it is also assumed that the observed enhancement in plant growth can be attributed to the ability of *P. indica* to modulate hormonal pathways as well as nutrient uptake.

The post-inoculation studies demonstrated that *P. indica* offers significant benefits in reducing the severity and impact of viral infections in bitter gourd, even when colonization occurs after the plants are infected. While disease incidence remained high, the reduction in disease severity, viral titers, and improvement in growth and yield parameters in *P. indica*-colonized plants emphasize its potential as a promising biocontrol agent in viral disease management. In the field studies, *P. indica* effectively reduced the incidence and severity of viral disease, delayed the appearance of viral symptoms, and enhanced overall plant growth and yield. From the pot culture as well as field studies, it can be inferred that if *P. indica*-colonized plants can be kept under protection for 15 days before exposure to the viruses, they would survive the viral infection and produce better yield. These results, combined with those from previous studies on *P. indica*, suggest its efficacy in integrated disease management strategies, especially against viral diseases in cucurbits and possibly also in other crops.

The interaction between the beneficial root endophyte *P. indica* and bitter gourd plants infected by BGMC has illustrated the complex biochemical and molecular mechanisms involved in the plant’s defense mechanisms. We have assessed the ROS and H_2_O_2_ accumulation, antioxidant enzyme activities, and the expression of important genes linked with them to elucidate how *P. indica* managed the viral diseases in bitter gourd. It was observed that the viral infection triggered the production and accumulation of ROS and H_2_O_2_ to cause the symptoms in plants. Interestingly, *P. indica* colonization suppressed the production and accumulation of ROS and H_2_O_2_ in the virus-infected plants.

One of the key findings from this study is the enhanced activity of antioxidant enzymes (catalase, superoxide dismutase, and peroxidase) in plants colonized by *P. indica*, particularly when inoculated with viruses. These enzymes play a crucial role in scavenging ROS, which tend to accumulate excessively in virus-infected plants, leading to oxidative stress and cellular damage. *Piriformospora indica* mitigated this response through the enhanced production of antioxidant enzymes to scavenge the excess ROS accumulation. Plants colonized by *P. indica* prior to the virus inoculation exhibited marginal ROS accumulation at 2 DPT, but these levels decreased over time (15 DPT and 30 DPT). This indicates that *P. indica* may initially trigger a controlled ROS burst, which is necessary for activating defense pathways, but subsequently reduces ROS accumulation to prevent tissue damage. The observed reduction in ROS levels in *P. indica*-colonized plants at later stages is consistent with the known ROS-scavenging role of *P. indica* reported by Waller et al. (2005), Serfling et al. (2007), Vadassery et al. (2009b), Sun et al. (2010), Johnson (2014), Matsuo et al. (2015), Sam (2021), and Sinijadas (2024). The reduced accumulation of ROS and H_2_O_2_ in the virus-inoculated plants colonized by *P. indica* at 15 DAI also confirms the ability of *P. indica* to modulate ROS even when colonization occurs after viral infection. The ability of *P. indica* to modulate oxidative stress responses (Hamilton et al., 2012; Matsuo et al., 2015) suggests that it could play a significant role in enhancing the resilience of bitter gourd plants to viral infections. By regulating ROS levels and improving antioxidant responses, *P. indica* contributes to improved plant health and yield, particularly in environments prone to viral diseases.

Interestingly, *P. indica* could simultaneously regulate ROS and H_2_O_2_ markers and antioxidant enzyme genes present in the nucleus, chloroplast, and mitochondria. While the endophyte significantly downregulates ROS and H_2_O_2_ marker genes located in the nucleus (*WRKY40*, *MYB51*, *CML37*, *AGP5*), chloroplast (*LOX2*, *PTOX*), and mitochondria (*HSPRO1*, *DIC2*, *PRX*), it upregulates ROS-scavenging genes encoding key antioxidant enzymes localized in the nucleus (*CSD1*, *APX1*), chloroplast (*FSD1*, *FSD2*), and mitochondria (*MSD1*, *AOX2*) to bring down the ROS to a level of absolute control, making the plant healthy and unstressed even in the presence of multiple viruses. The multiple virus infection led to the upregulation of ROS and H_2_O_2_ marker genes and downregulation of antioxidant genes in different organelles. This led to excess production and accumulation of ROS and H_2_O_2_, which results in the weakening of plant cells and symptom development. The ability of *P. indica* to regulate genes present in the nucleus, chloroplast, and mitochondria through the retrograde signaling molecule(s) generated by the *P. indica*–plant interaction system with abiotic and biotic stress was reported by Vadassery et al. (2009a), Sun et al. (2010), Johnson et al. (2014b, 2018).

To summarize, our findings demonstrate that *P. indica* confers significant protection against the bitter gourd mosaic complex by orchestrating the host’s oxidation–reduction processes, reducing viral titer, and enhancing plant growth, vigor, and yield. Together, these effects mitigate cytotoxic damage to improve photosynthetic efficiency and ultimately enhance plant growth and yield. Gene expression analyses further revealed that *P. indica* colonization in virus-infected plants modulated the expression of redox signaling–related genes in the nucleus, chloroplast, and mitochondria. The results also show simultaneous downregulation of ROS marker genes and upregulation of antioxidant genes located in the nucleus, chloroplast, and mitochondria of leaves of colonized plants when infected with viruses. This clearly indicates the role of *P. indica* in orchestrating the regulation of ROS production and its scavenging through systemic retrograde signaling molecule(s) produced by the root endophyte. This study presents the first salient report of *P. indica* effectively managing multiple viral infections in plants. *Piriformospora indica* has an exceptional ability to simultaneously control three distinct viruses with different genome organizations (DNA, RNA) and from three different families, namely, *Geminiviridae* (ToLCV), *Potyviridae* (PRSV), and *Bromoviridae* (CMV) under both controlled and field conditions. The fungus thus represents a promising, eco-friendly tool for managing complex viral infections in cucurbits as well as other crops. Furthermore, the study warrants the detailed profiling of metabolomes, proteomes, and transcriptomes that are specifically induced or repressed to understand other mechanisms involved in the tripartite interaction of plant, endophytes, and viruses.

The compatibility and performance of *P. indica* with promising bacterial endophytes that promote plant growth and confer multiple pathogen tolerance, such as *Bacillus amyloliquefaciens* (Sharmila et al., 2025), *Bacillus subtilis* (Mageshwaran et al., 2022; Taheri et al., 2023), and *Pseudomonas fluorescens* (Sharmila *et al*., unpublished), need to be explored. Large-scale on-farm field evaluations of *P. indica* in different crops under diverse agro-ecological units/zones warrant the commercial use of this endophyte for sustainable crop production and protection. Identification and characterization of the systemic signaling molecule(s) of *P. indica*, which are produced in roots and modulate different biochemical, physiological, and molecular responses in the shoots, will generate a lot of academic interest.

## Data Availability

The datasets presented in this study can be found in online repositories. The names of the repository/repositories and accession number(s) can be found in the article/Supplementary material.
