# Supplementary material for: Beneficial fungal root endophyte Piriformospora indica inhibits bitter gourd mosaic complex disease incited by combined infection of tomato leaf curl, papaya ringspot, and cucumber mosaic viruses without compromising crop growth and yield by orchestrating ROS production and scavenging through retrograde signaling
Source: Front Microbiol. 2026 Mar 18;17:1781341. doi: 10.3389/fmicb.2026.1781341 (PMC13038624; doi:10.3389/fmicb.2026.1781341)
Supplement: Supplementary file 1 [file Data_Sheet_1.PDF]

Supplementary Materials

1. Supplementary Tables

Supplementary Table S1. Details of the coat protein primers used for the molecular detection of ToLCV, PRSV and CMV in bitter gourd plants

Virus	Primer name	Sequences	Size (bp)	Reference
ToLCV (*Begomo-virus*)	Deng 541 F	TAATATTACCKGWKGVCCSC	520	Deng *et al*., 1994
	Deng 540 R	TGGACYTTRCAWGGBCCTTCACA		
	AV 494 F	GCCHATRTAYAGRAAGCCMAGRAT	575	Wyatt and Brown, 1996
	AC 1048 R	GGRTTDGARGCATGHGTACANGCC		
	GK ToLCV F	ATGKYGAAGCGACCAGCMGA	950	Nagendran *et al*., 2014
	GK ToLCV R	CGCCCKCMGAYTGGG MTTTTCTT		
PRSV (*Potyvirus*)	GK PRSV F	GCAATGATAGARTCATGGGG	1267	Nagendran *et al*., 2017
	GK PRSV R	AAGCGGTGGCGCAGCCACACT		
CMV (*Cucumovirus*)	GK CMV F	GAGTTCTTCCGCGTCCCGCT	1218	Nagendran *et al*., 2017
	GK CMV R	AAACCTAGGAGATGGTTTCA		

Supplementary Table S2. Details of the primers used for the gene expression studies using qRT-PCR

Sl. No.	Gene	Primers		Length	Tm (°C)	Amplicon size (bp)
1	*WRKY40*	LP	GCTTGGTTGTGAAAGATGGGT	21	59.0	107
		RP	ACAATTTGGTGCAGAGGAGC	20		
2	*MYB51*	LP	GGACTCCCGAGGAAGATCAG	20	59.0	104
		RP	TTTGCCGCATCTCTTGAGTC	20		
3	*CML37*	LP	GTTTATTACGGCGGCGAGTT	20	58.5	119
		RP	AGCTGAGAACTCCATCGTCA	20		
4	*AGP5*	LP	ACCGTCTCAATCGCCTAGAG	20	59.0	136
		RP	GGAAGTAGACGTCGGAGGAG	20		
5	*LOX2*	LP	GCTGTTGACGGGTTGGAAAT	20	59.0	112
		RP	AGGCTTGGAGTTCTGCATCT	20		
6	*PTOX*	LP	TCGGAATGTGTGGAGAGTCA	20	58.0	128
		RP	AAGTACAAGTCACCTCCCGT	20		
7	*HSPR01*	LP	TACCTCCGTTTGCCTGAACT	20	59.0	134
		RP	TCAGAAGCAACGGTGAGGAT	20		
8	*DIC2*	LP	AATGTCGAAGCAGGAGGGAA	20	59.0	144
		RP	CCGTCCTTCATCACTCCCTT	20		
9	*PRX*	LP	TTGCTCTTGTTGGTGCTCAC	20	59.0	136
		RP	GCACACAACTTCCTCAGCTC	20		
10	*CSD1*	LP	TGCCACCTTCACAATCACTG	20	58.0	150
		RP	TGAGTTCATGGCCTCCCTTT	20		
11	*APX1*	LP	GGGTCGTCTTCCTGATGCTA	20	59.0	138
		RP	CCAGACCTATCCTTGTGGCA	20		
12	*FSD1*	LP	GGTTCTTGGAACCGAGCTTG	20	59.0	127
		RP	CTCCCAGAAGAACTCGTGGT	20		
13	*FSD2*	LP	CCACGAGTTCTTCTGGGAGT	20	59.0	125
		RP	CAGCCGACTTGAACCTTTCC	20		
14	*MSD1*	LP	CATTGACGCTCACTTTGGCT	20	59.0	103
		RP	TTTGTCTAGTCCGAGCCACA	20		
15	*AOX2*	LP	AGCTTCTTCGGATCCCAACA	20	58.5	146
		RP	CCCACTGTGCTGAAACTTCC	20		
16	*GAPDH*	LP	CTAGAGTTGTTCTCCAGAGGGAC	23	61.0	122
		RP	CAAGGCAGTTAGTGGTGCAGCTA	23		

Supplementary Table S3. Specific symptoms produced by individual virus and its combinations in bitter gourd mosaic complex disease (Chandran *et al*., 2024)

Viruses and its combinations	Specific symptoms produced in bitter gourd
ToLCV (*Begomovirus*)	Upward leaf curling and mosaic, hairiness
PRSV (*Potyvirus*)	Blisters and puckering on leaves, yellowing of leaves with downward curling
CMV (*Cucumovirus*)	Mottling of leaves, leaf distortion
ToLCV and PRSV	Upward leaf curling, blistering and puckering on leaves
ToLCV and CMV	Upward leaf curling, leaf mottling and leaf distortion
PRSV and CMV	Downward leaf curling and yellowing, puckering of leaves with or without distortion
ToLCV, PRSV and CMV	Upward leaf curling, blistering and puckering, leaf distortion, hairiness and stunted growth

Supplementary Table S4. Immunological detection of ToLCV, PRSV and CMV in bitter gourd mosaic complex samples using DAS-ELISA and DIBA

Plant sample	DAS-ELISA (OD value at 405 nm*)			DIBA (Mean intensity value*)		
	ToLCV	PRSV	CMV	ToLCV	PRSV	CMV
1	0.078	0.092	0.063	1794.4	2594.2	1638.1
2	0.042	0.102	0.062	1565.4	2632.6	1502.7
3	0.082	0.078	0.048	2301.5	2297.3	1686.4
4	0.055	0.066	0.043	1908.9	1598.3	1559.2
5	0.046	0.081	0.067	1668.2	1689	1572.8
Healthy	0.004	0.007	0.006	388.5	458.9	360.5
Buffer	0.003	0.004	0.004	254.8	395.1	336.9

The diseased samples were collected from the fields showing the characteristic symptoms of BGMC disease and tested for the presence of PRSV, ToLCV and CMV using the virus-specific polyclonal antibodies purchased from Leibniz Institute DSMZ-German Collection of Microorganisms and Cell Cultures GmbH, Germany. Values are mean of three replications.

Supplementary Table S5. Immunological detection of ToLCV, PRSV and CMV by DAS-ELISA in *P. indica*-colonized bitter gourd plants infected with the viruses inciting bitter gourd mosaic complex disease in the pre-inoculation studies

Treatments	OD value at 405 nm *								
	15 DAI			45 DAI			75 DAI		
	ToLCV	PRSV	CMV	ToLCV	PRSV	CMV	ToLCV	PRSV	CMV
+Pi/+V_0	0.072	0.074	0.081	0.042	0.060	0.072	0.040	0.052	0.050
+Pi/+V_2	0.056	0.068	0.073	0.036	0.048	0.056	0.036	0.046	0.046
+Pi/+V_5	0.058	0.062	0.068	0.032	0.043	0.047	0.032	0.032	0.035
+Pi/+V_{10}	0.042	0.038	0.046	0.023	0.033	0.041	0.024	0.027	0.026
+Pi/+V_{15}	0.032	0.042	0.032	0.019	0.020	0.028	0.019	0.023	0.019
+V alone	0.109	0.112	0.109	0.084	0.111	0.098	0.085	0.084	0.085
+Pi alone	0.007	0.006	0.007	0.006	0.007	0.005	0.006	0.005	0.007
Absolute Control	0.008	0.007	0.008	0.005	0.006	0.006	0.006	0.007	0.007

P. indica-colonized bitter gourd seedlings (4 leaf-stage) were graft-transmitted with scions having ToLCV, PRSV and CMV at 0 (+Pi/+V_0), 2 (+Pi/+V_2), 5 (+Pi/+V_5), 10 (+Pi/+V_{10}) and 15 (+Pi/+V_{15}) days after the colonization as described in the materials and methods. The control seedlings (without *P. indica*) wedge grafted with the viruses-infected scion was positive control (+V alone) and *P. indica*-colonized seedlings without the viruses served as negative control (+Pi alone). The seedlings without any treatment served as absolute control. Five plants from each treatment were randomly taken. * Values are mean of 5 replications. Five independent experiments were done. DAI: days after inoculation of virus by grafting.

Supplementary Table S6. Immunological detection of ToLCV, PRSV and CMV by DAS-ELISA in *P. indica*-colonized bitter gourd plants infected with bitter gourd mosaic complex disease in the post-inoculation studies

Treatments	OD value at 405 nm *								
	15 DAC			45 DAC			75 DAC		
	ToLCV	PRSV	CMV	ToLCV	PRSV	CMV	ToLCV	PRSV	CMV
+V/+Pi$_0$	0.082	0.064	0.084	0.070	0.079	0.042	0.050	0.068	0.040
+V/+Pi$_2$	0.084	0.070	0.082	0.078	0.085	0.052	0.052	0.072	0.045
+V/+Pi$_5$	0.088	0.072	0.081	0.082	0.090	0.054	0.054	0.075	0.051
+V/+Pi$_{10}$	0.106	0.086	0.116	0.098	0.100	0.054	0.070	0.086	0.060
+V/+Pi$_{15}$	0.110	0.088	0.119	0.102	0.108	0.064	0.075	0.091	0.066
+V alone	0.129	0.090	0.139	0.138	0.128	0.084	0.092	0.108	0.096
+Pi alone	0.007	0.007	0.007	0.005	0.008	0.005	0.006	0.005	0.006
Absolute Control	0.008	0.007	0.009	0.008	0.008	0.006	0.006	0.007	0.007

The viruses-infected scions (ToLCV, PRSV and CMV) were graft-transmitted to 4 leaf-stage healthy bitter gourd seedlings grown in pots filled with potting mixture; and then the viruses-inoculated-seedlings were transplanted to pots filled with *P. indica*-multiplied potting mixture having 10^6 cfu g^{-1} for the fungal colonization at 0 (+V/+Pi$_0$), 2 (+V/+Pi$_2$), 5 (+V/+Pi$_5$), 10 (+V/+Pi$_{10}$) and 15 (+V/+Pi$_{15}$) days after the viruses inoculation as described in the materials and methods. The control seedlings (without *P. indica*) wedge grafted with the viruses-infected scion was positive control (+V alone) and *P. indica*-colonized seedlings without the viruses served as negative control (+Pi alone). The seedlings without any treatment served as absolute control. Five plants from each treatment were randomly taken. * Values are mean of 5 replications. Five independent experiments were carried out. DAC: days after colonization of *P. indica*.

Supplementary Table S7. Immunological detection of ToLCV, PRSV and CMV in *P. indica*-colonized bitter gourd plants by DAS-ELISA under natural incidence of bitter gourd mosaic complex disease in field condition during summer season

Treatments	OD value at 405 nm *											
	0 DAT			15 DAT			45 DAT			75 DAT		
	ToLCV	PRSV	CMV	ToLCV	PRSV	CMV	ToLCV	PRSV	CMV	ToLCV	PRSV	CMV
+ *P. indica*	0.006	0.010	0.003	0.004	0.011	0.004	0.022	0.028	0.021	0.018	0.036	0.032
Control	0.008	0.009	0.002	0.028	0.045	0.023	0.036	0.053	0.037	0.039	0.075	0.045
Healthy	0.008	0.007	0.002	0.005	0.006	0.006	0.006	0.009	0.009	0.006	0.007	0.002

Fifteen-day-old *P. indica*-colonized and control bitter gourd seedlings, grown as per the procedure mentioned in materials and methods, were transplanted to the pits @ 3 seedlings per pit for both treatments. * Values are mean of three replications. Three independent experiments were done.

Supplementary Table S8. Immunological detection of ToLCV, PRSV and CMV in *P. indica*-colonized bitter gourd plants by DAS-ELISA under natural incidence of bitter gourd mosaic complex disease in field condition during rabi season

Treatments	OD value at 405 nm *											
	0 DAT			15 DAT			45 DAT			75 DAT		
	ToLCV	PRSV	CMV	ToLCV	PRSV	CMV	ToLCV	PRSV	CMV	ToLCV	PRSV	CMV
+ *P. indica*	0.010	0.005	0.003	0.004	0.001	0.005	0.018	0.018	0.020	0.024	0.036	0.028
Control	0.007	0.002	0.003	0.039	0.025	0.003	0.043	0.043	0.036	0.042	0.055	0.045
Healthy	0.006	0.009	0.005	0.005	0.003	0.004	0.004	0.007	0.009	0.006	0.006	0.007

Fifteen-day-old *P. indica*-colonized and control bitter gourd seedlings, grown as per the procedure mentioned in materials and methods, were transplanted to the pits @ 3 seedlings per pit for both treatments. * Values are mean of three replications. Three independent experiments were done.

2. Supplementary Figures

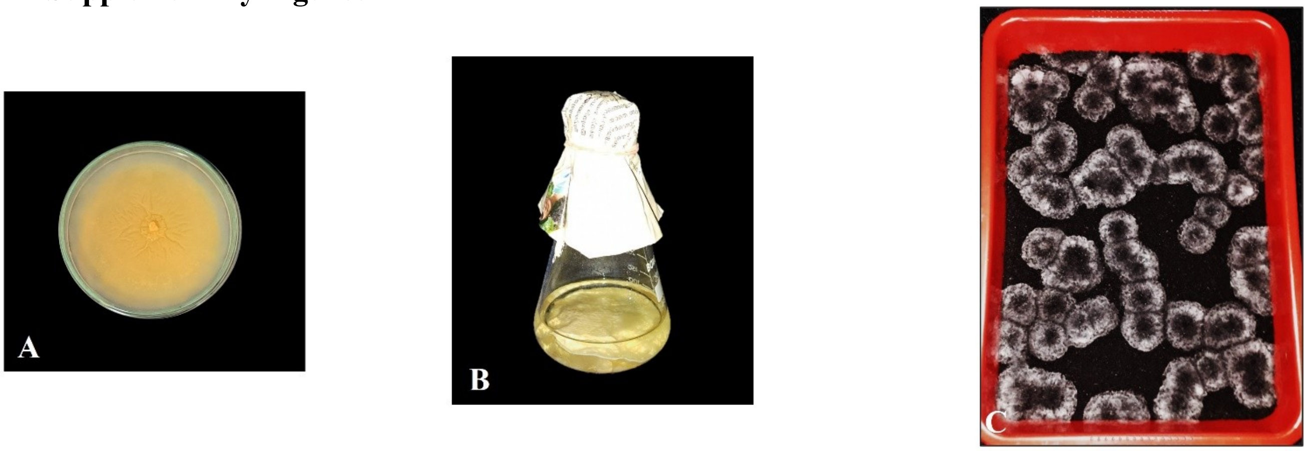

Supplementary Figure 1. Mass-multiplication of *P. indica* on coco peat-farm yard manure medium - (A) *P. indica* in PDA medium at 14 DAI, (B) *P. indica* in PDB at 14 DAI, (C) Mycelial growth of *P. indica* in coco peat-FYM-gram flour (1:1:0.02) medium at 5 DAI (DAI - Days after inoculation).

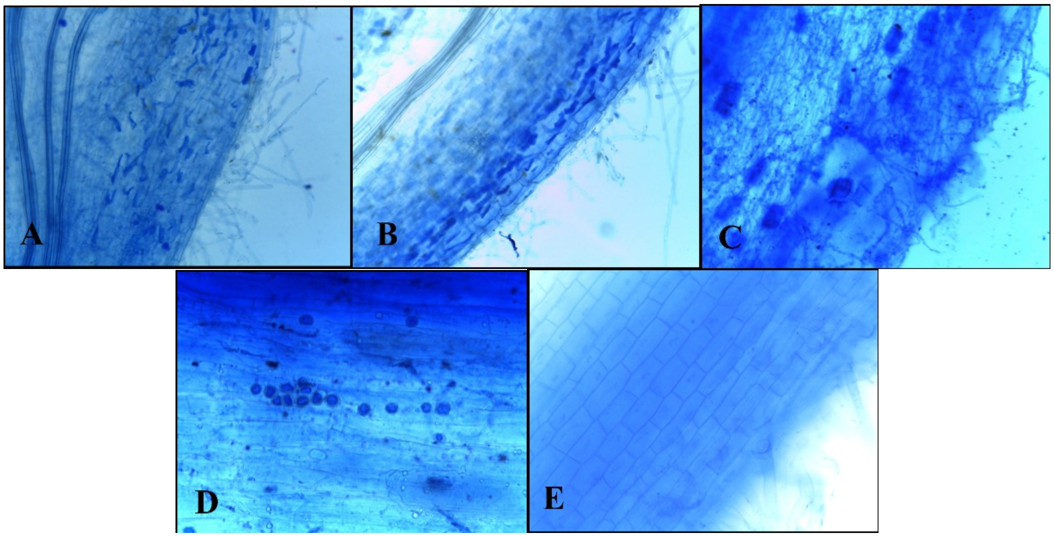

Supplementary Figure 2. Microscopic image of *P. indica*-colonized root section of bitter gourd seedlings (A) mycelial growth in and on the roots at 3 DAG, (B) mycelial growth in and on the roots at 5 DAG, (C) chlamydospores formation inside the root at 10 DAG, (D) chlamydospores inside the root at 15 DAG; and (E) control plant at 400X magnification.

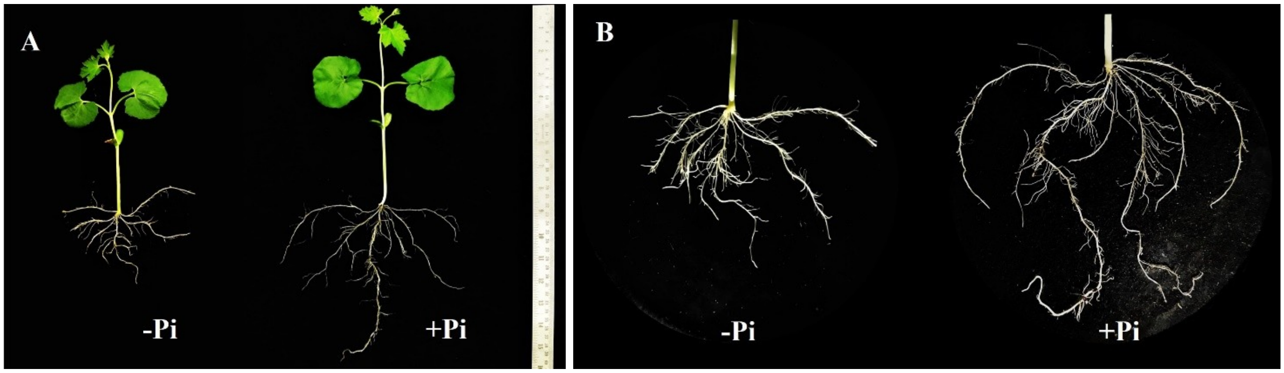

Supplementary Figure 3. *P. indica*-colonization promotes the growth of bitter gourd seedlings after 5 days of germination. Seedling, shoot and root growth (A); Root growth with secondary and tertiary roots (B). The seeds were sown in *P. indica*-mass multiplied cocopeat-FYM-gram flour medium as described in materials and methods. 10 replications per treatment were maintained. Five independent experiments were done. Representative pictures are shown.

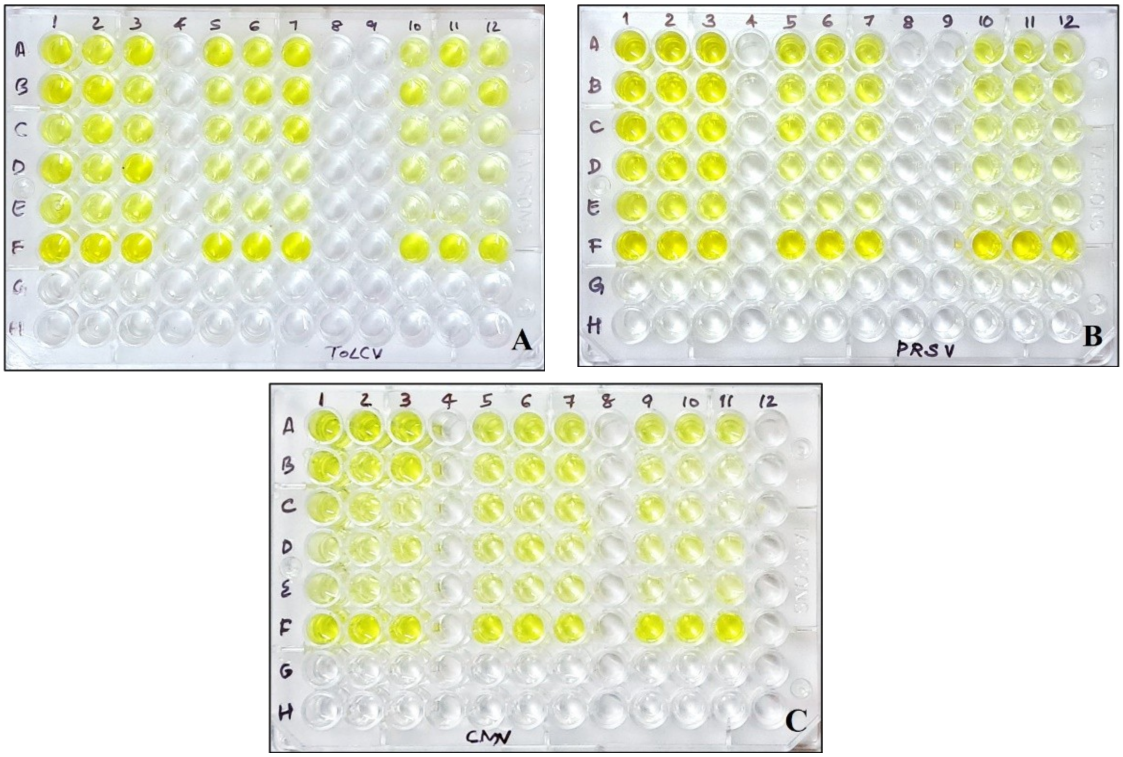

Well number	Sample at 15 DAI	Well number	Sample at 45 DAI	Well number	Sample at 75 DAI
1A – 3A	$+Pi+V_0$	5A – 7A	$+Pi+V_0$	10A – 11A	$+Pi+V_0$
1B – 3B	$+Pi+V_2$	5B – 7B	$+Pi+V_2$	10B – 11B	$+Pi+V_2$
1C – 3C	$+Pi+V_5$	5C – 7C	$+Pi+V_5$	10C – 11C	$+Pi+V_5$
1D – 3D	$+Pi+V_{10}$	5D – 7D	$+Pi+V_{10}$	10D – 11D	$+Pi+V_{10}$
1E – 3E	$+Pi+V_{15}$	5E – 7E	$+Pi+V_{15}$	10E – 11E	$+Pi+V_{15}$
1F – 3F	+V alone	5F – 7F	+V alone	10F – 11F	+V alone
1G – 3G	+Pi alone	5G – 7G	+Pi alone	10G – 11G	+Pi alone
1H – 3H	Control	5H – 7H	Control	10H – 11H	Control
8F – 8H			Buffer		

Supplementary Figure 4. Serological detection and titre of the viruses causing bitter gourd mosaic complex by DAS-ELISA from the pre-inoculation studies (A) ToLCV, (B) PRSV and (C) CMV at 15 DAI, 45 DAI and 75 DAI. *P. indica*-colonized bitter gourd seedlings (4 leaf-stage) were graft-transmitted with scions having ToLCV, PRSV and CMV at 0 ($+Pi/+V_0$), 2 ($+Pi/+V_2$), 5 ($+Pi/+V_5$), 10 ($+Pi/+V_{10}$) and 15 ($+Pi/+V_{15}$) days after the colonization as described in the materials and methods. The control seedlings (without *P. indica*) wedge-grafted with the viruses-infected scion was positive control (+V alone) and *P. indica*-colonized seedlings without the viruses served as negative control (+Pi alone). The seedlings without any treatment served as absolute control. Three plants from each treatment were randomly taken. Five independent experiments were done. DAI: days after inoculation of virus by grafting. Yellow colour - positive to virus, colourless - negative to virus.

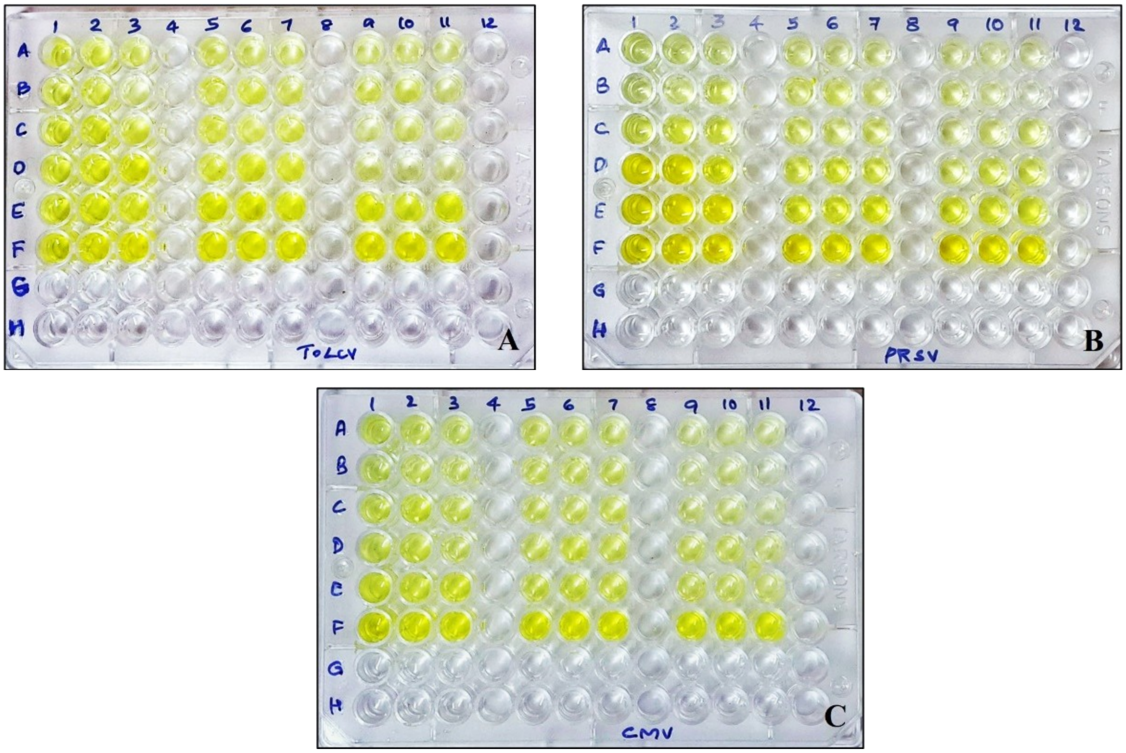

Well number	Sample at 15 DAC	Well number	Sample at 45 DAC	Well number	Sample at 75 DAC
1A – 3A	+V/+Pi_0	5A – 7A	+V/+Pi_0	10A – 11A	+V/+Pi_0
1B – 3B	+V/+Pi_2	5B – 7B	+V/+Pi_2	10B – 11B	+V/+Pi_2
1C – 3C	+V/+Pi_5	5C – 7C	+V/+Pi_5	10C – 11C	+V/+Pi_5
1D – 3D	+V/+Pi_{10}	5D – 7D	+V/+Pi_{10}	10D – 11D	+V/+Pi_{10}
1E – 3E	+V/+Pi_{15}	5E – 7E	+V/+Pi_{15}	10E – 11E	+V/+Pi_{15}
1F – 3F	+V alone	5F – 7F	+V alone	10F – 11F	+V alone
1G – 3G	+Pi alone	5G – 7G	+Pi alone	10G – 11G	+Pi alone
1H – 3H	Control	5H – 7H	Control	10H – 11H	Control
8F – 8H			Buffer		

Supplementary Figure 5. Serological detection and titre of the viruses causing bitter gourd mosaic complex by DAS-ELISA from the post-inoculation studies (A) ToLCV, (B) PRSV and (C) CMV at 15 DAI, 45 DAI and 75 DAI. The viruses-infected scions (ToLCV, PRSV and CMV) were graft-transmitted to 4 leaf-stage healthy bitter gourd seedlings grown in pots filled with potting mixture; and then the viruses-inoculated-seedlings were transplanted to pots filled with *P. indica*-multiplied potting mixture having 10^6 cfu g^{-1} for the fungal colonization at 0 (+V+Pi_0), 2 (+V+Pi_2), 5 (+V+Pi_5), 10 (+V+Pi_{10}) and 15 (+V+Pi_{15}) days after the viruses inoculation as described in the materials and methods. The control seedlings (without *P. indica*) wedge grafted with the viruses-infected scion was positive control (+V alone) and *P. indica*-colonized seedlings without the viruses served as negative control (+Pi alone). The seedlings without any treatment served as absolute control. Three plants from each treatment were randomly taken. Five independent experiments were done. DAC: days after colonization of *P. indica*. Yellow colour - positive to virus, colourless - negative to virus.

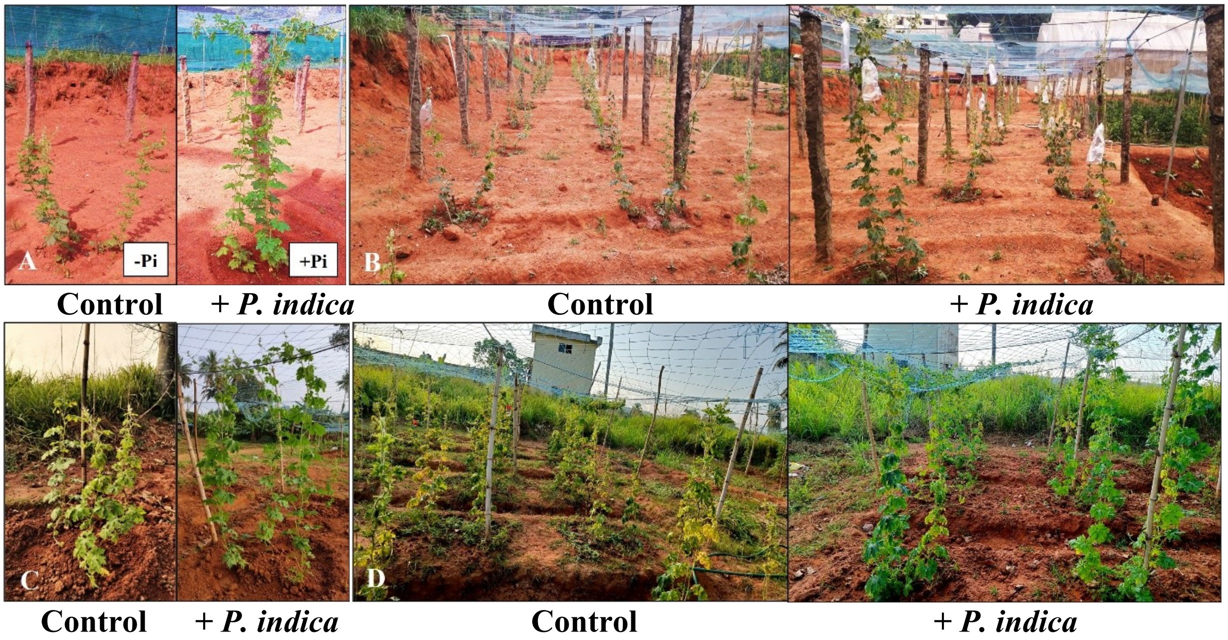

Supplementary Figure 6. Field view of studies on natural incidence of bitter gourd mosaic complex in *P. indica*-colonized (+Pi) and control plants (-Pi) during summer season at 30 DAT (A), and 60 DAT (B); rabi season at 30 DAT (C), and 60 DAT (D). Fifteen-day-old *P. indica*-colonized and control bitter gourd seedlings, grown as per the procedure mentioned in materials and methods, were transplanted to the pits @ 3 seedlings per pit for both treatments. Three independent experiments per season were done. Representative pictures are shown.

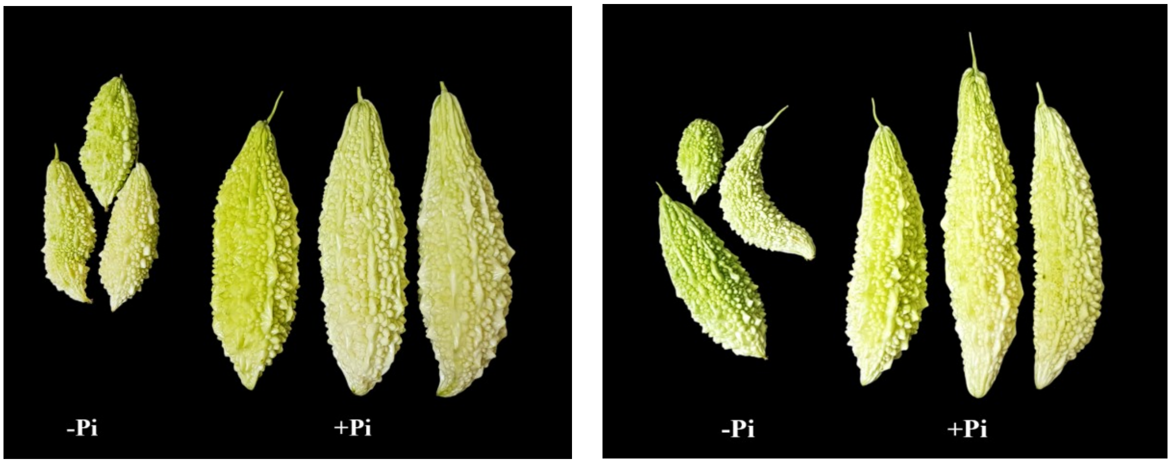

A B

Supplementary Figure 7. Fruits of *P. indica*-colonized (+Pi) and control plants (-Pi) from field studies on natural incidence of bitter gourd mosaic complex during (A) summer and (B) rabi seasons. Fifteen-day-old *P. indica*-colonized and control bitter gourd seedlings, grown as per the procedure mentioned in materials and methods, were transplanted to the pits @ 3 seedlings per pit for both treatments. Three independent experiments per season were done. Representative pictures are shown.

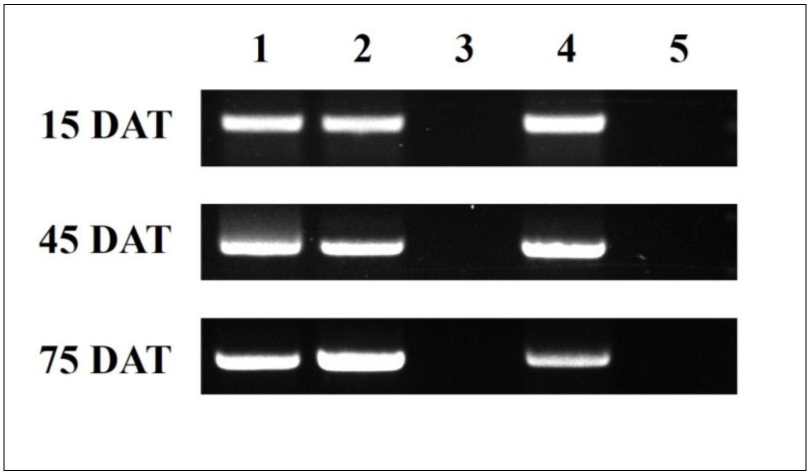

Lane 1: +Pi +$V_{15,}$ Lane 2: +V +$Pi_{15,}$ Lane 3: +V, Lane 4: +Pi, Lane 5: absolute control.

Supplementary Figure 8. Electrophoresis gel image of amplicon of *Pi-tef* gene detected in bitter gourd roots of *P. indica*-colonized plants on pre- and post- inoculation of ToLCV, PRSV and CMV causing bitter gourd mosaic complex at 2 DPT, 15 DPT and 30 DPT. *P. indica*-colonized bitter gourd seedlings (4 leaf-stage) were graft-transmitted with scions having the viruses at 15 days after the colonization (+Pi/+V_{15}; Lane 1). The viruses-infected scions were graft-transmitted to 4 leaf-stage healthy bitter gourd seedlings grown in pots filled with potting mixture; and then the viruses-inoculated-seedlings were transplanted to pots filled with *P. indica*-multiplied potting mixture having 10^6 cfu g^{-1} for the fungal colonization at 15 days after the viruses inoculation (+V+Pi_{15}; Lane 2). The control seedlings (without *P. indica*) wedge grafted with the viruses-infected scion was positive control (+V; Lane 3) and *P. indica*-colonized seedlings without the viruses served as negative control (+Pi; Lane 4). The seedlings without any treatment served as control (-PI-V; Lane 5). Ten plants per treatment. Five independent experiments were done.
